# Analysis of content and online public responses to media articles that raise awareness of the opt-out system of consent to organ donation in England

**DOI:** 10.3389/fpubh.2022.1067635

**Published:** 2022-12-01

**Authors:** Georgia Faherty, Lorraine Williams, Jane Noyes, Leah Mc Laughlin, Jennifer Bostock, Nicholas Mays

**Affiliations:** ^1^Policy Innovation and Evaluation Research Unit (PIRU), Department of Health Services Research and Policy, London School of Hygiene and Tropical Medicine, London, United Kingdom; ^2^School of Medical and Health Sciences, Bangor University, Bangor, United Kingdom

**Keywords:** organ donation, public opinion, consent, media campaigns, soft opt-out, media content analysis

## Abstract

**Background:**

Preceded by a national media campaign, in May 2020, England switched to a soft opt-out system of organ donation which rests on the assumption that individuals meeting specific criteria have consented to organ donation unless they have expressed otherwise. We aimed to learn more about how the changes were communicated, how people responded and any discrepancies between key messages and how they were interpreted by the public.

**Methods:**

Summative content analysis of 286 stories and related reader-generated comments in leading UK online news sources (April 2019 to May 2021). Further detailed thematic analysis of 21 articles with reader-generated content, complemented by thematic content analysis coding of all 286 stories.

**Results:**

Most media coverage on both organ donation and the law change was positive, with little variation over time or between publications. The importance of organ donation, benefits of the law change, and emotive stories (often involving children) of those who had donated an organ described as “superheroes” or those who had received organs as benefiting from a “miracle” were frequently cited. In contrast, reader-generated comments were markedly more negative, for example, focusing on loss of individual freedom and lack of trust in the organ donation system. Commentators wished to be able to choose who their organs were donated to, were dismissive and blaming towards minority ethnic groups, including undermining legitimate worries about the compatibility of organ donation with religious beliefs and end of life cultural norms, understanding and acceptance of brain-stem death and systemic racism. Misinformation including use of inflammatory language was common.

**Conclusion:**

The portrayal of donors and recipients as extraordinary is unlikely to help to normalise organ donation. Undermining legitimate concerns, in particular those from ethnic minorities, can alienate and encourage harmful misinformation in underrepresented groups. The discrepancies between the tone of the articles and the readers comments suggests a lack of trust across the public, health, policy and media outlets. Easily accessible, ongoing and tailored sources are needed to mitigate misinformation and disinformation and ensure key messages are better understood and accepted in order to realise the ambitions of soft opt-out organ donation policies.

## Introduction

Increasing the number of organs available for life saving transplants remains a global health priority (1). In healthcare systems with established organ donation and transplant programmes, interventions designed to increase the number of organs available are extensive, ranging from developing technologies to better use organs when they become available (living and deceased) to ongoing media campaigns to promote awareness and influence behaviour (2, 3). Since 2008 the United Kingdom (UK) has been implementing system wide interventions designed to address organ shortages (4). One such intervention is switching to an ‘opt-out' system of organ donation (5). Versions of opt-out systems vary but the underpinning principles are the same—an opt-out system of organ donation switches the default position of citizens to one that positively supports organ donation (6). Over the past 30 years or so countries with developed healthcare systems are increasingly adopting opt-out policies (7), despite evidence suggesting that taken in isolation it is difficult to unpick the impacts of legislation on consent rates or number of transplants (8).

In May 2020, England switched to a ‘soft' opt-out system of organ donation, one which requires consultation and support from families of the deceased (9). Here people who meet specific criteria are presumed to support organ donation unless they have expressed otherwise during their lifetime (10) (Box 1).

Box 1 **Summary of the opt-out legislation (England) and who it affects.**

All citizens over 18 years that have mental capacity to understand the changes and have lived in England for at least 12 months before their death are presumed to have consented to organ donation after they die, unless they have explicitly registered on the organ donor register or verbally expressed a decision not to do so. Families are now expected to put their own views aside and support the decision their relative made while they were alive regarding organ donation.

Implementing population health policy changes such as this are complex and often rely heavily on public media communications and carefully crafted messages delivered through multiple channels (11). When Wales (a devolved country within the UK) switched to a very similar system, media tone towards organ donation and the new policy changed—becoming more positive after implementation over time (12). Previous research has demonstrated that the topic of organ donation is newsworthy but when linked with controversial narratives will be given more prominence (e.g., front page headlines) (13) and that, as well as playing an important role in communicating information about organ donation, local and national media can both reflect *and* influence public opinion (12).

The role of the media and the ways the general public consume messages has changed dramatically in recent years with online news and social media including ‘influencers' playing an increasingly important role in engaging audiences, communicating information, and changing attitudes and behaviour (14). Understanding how the public respond to and interpret health messages is becoming increasingly important as reader-generated content becomes a common source of news in itself (15). Recently the World Health Organisation argued that misinformation (propagated by reader-generated content) was potentially as great a source of harm as the COVID-19 virus to public health (16). Research to investigate how health messages are both communicated and understood is vital in understanding how to prevent spread of mis- and dis-information, and in developing future communication strategies that make it possible for people to find trustworthy sources and reliable guidance when they need it.

National Health Service Blood and Transplant (NSHBT), the organisation responsible for providing organ donation and transplant services in the UK, and its communications team developed a national comprehensive media strategy and organ donation campaign “Pass it On” (17). Launched on the 25 April 2019, it aimed to communicate the change in the law and at the same time promote organ donation as an act of gifting (18). The messages were designed to set a positive tone, positively frame narratives and stories resulting from the law change, and encourage the public (as potential donors and family members) to accept the changes and talk about organ donation (19). The campaign was timed for 1 year before implementation with targeted “bursts” of activity planned throughout the implementation phases (20).

In this study we aimed to identify the tone (positive, negative, neutral) of the media coverage related to organ donation and associated reader-generated comments in the year leading up to the implementation of the ‘soft opt-out' system of organ donation in England and the 12 months after implementation.

## Materials and methods

### Aim and objectives

To undertake an analysis of media articles and associated online public responses about organ donation and the soft opt-out system of organ donation in the year leading up to the change and 1 year afterwards (20 May 2019 to 20 May 2021) in order to explore:

- *How the law change was communicated by news media*; including whether the tone was positive, neutral, or negative, how the change was framed, whether the reporting was accurate, and whether there were differences between publications, or over time;
- *How the public responded*; whether the change was well-understood, whether reactions were positive, neutral or negative, and whether there were differences between readers of different publications, or over time;
- *Any differences in how issues relating to organ donation were presented by policy makers, communicated by the media, and discussed by readers;* and the possible implications of this for the relationship between these groups;
- *Other themes relating to public understanding and attitudes to the change to a soft op out system;* including how these may have been affected by the COVID-19 pandemic.

### Methods

**Figure 1:**
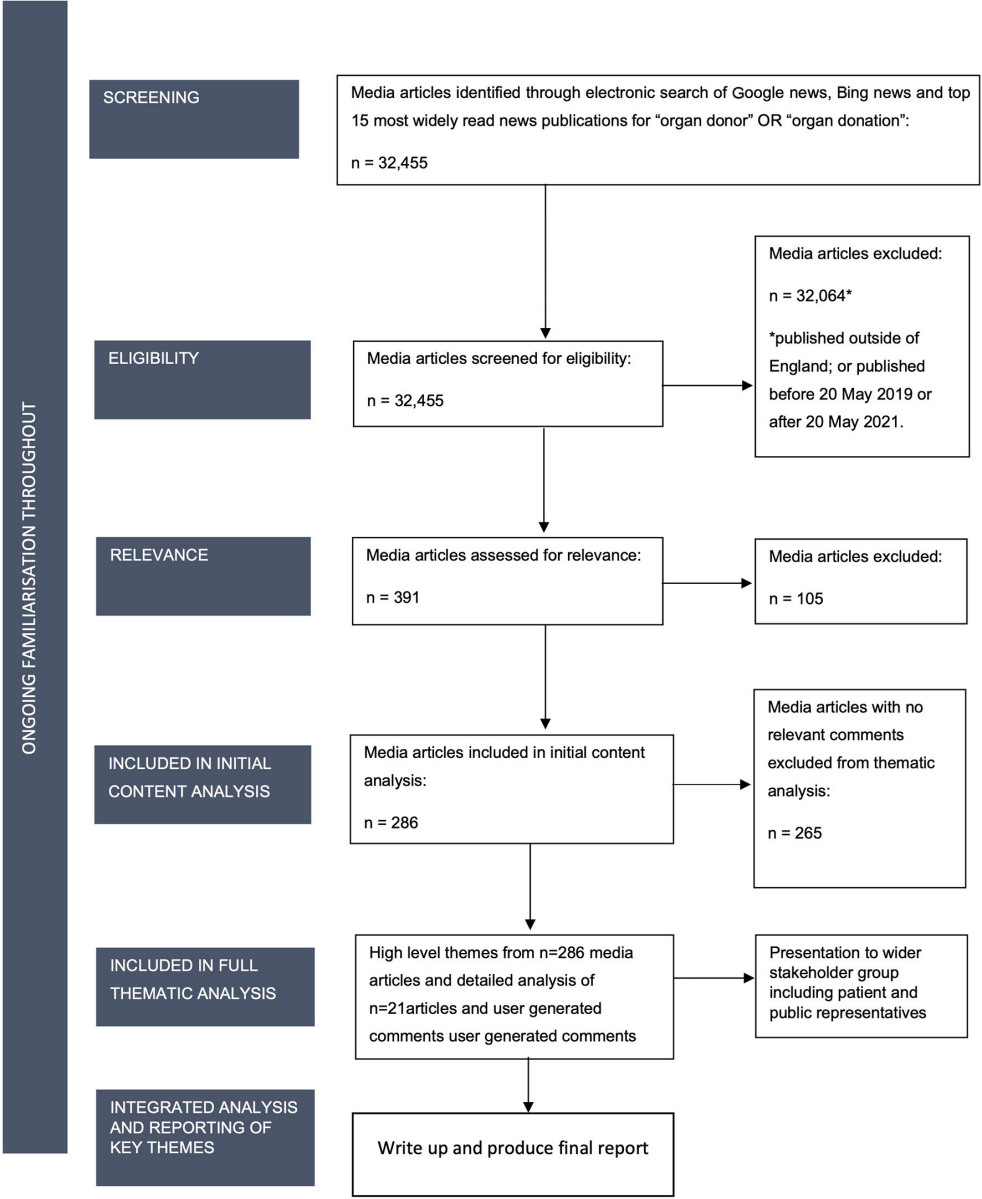
Search strategy.

We used Powell and van Velthoven's guidance on collecting and analysing digital data (21). Two methods of analysis were used—summative content analysis (22) and thematic analysis (23). The stages are presented in Figure 1 and described in further detail below (Figure 1).

#### Search strategy

A purposive sampling approach was used to identify news media articles and reader-generated comments available online that referred to organ donation and were published between 20 May 2019 and 20 May 2021. Media sources were identified according to their reach and readership. Google news, Bing news and 15 individual news websites with the highest levels of readership in England (Appendix 1) were searched for the terms ‘organ donor' OR “organ donation.” The initial search returned 32,455 results. After the exclusion of articles which did not meet the inclusion criteria and duplicate articles, 286 remained.

##### Inclusion and exclusion criteria

Articles were excluded from initial summative content analysis if they did not meet the following inclusion criteria:

- Published between 20 May 2019 and 20 May 2021. These dates were chosen to include coverage for 1 year before the law change, and 1 year after its implementation. The dates also align closely with NHSBTs media campaign, ‘Pass it on,' which was launched on 25 April 2019.
- Published by news media organisations based in England and aimed at an English audience.
- Contained relevant subject matter relating to deceased organ donation and/or the change in the law in England.

#### Data analysis

Once all relevant articles that met the inclusion criteria were identified, pdf copies were obtained and downloaded, and then imported into NVivo software (v12) for further analysis (24). Articles were labelled by the date and newspaper of publication. As the articles were downloaded and imported, they were read again to ensure the article was relevant, to become more familiar with the content, and to make initial observations.

##### Summative content analysis

Initial summative content analysis was undertaken for all 286 articles including the 21 articles which had accompanying reader-generated comments (Appendix 2) (22). Each article and any relevant reader-generated comments were reviewed and coded according to whether their tone was interpreted by the researcher as positive, neutral, or negative in relation to organ donation, and separately whether the tone was interpreted as positive, neutral, or negative in relation to the law change. In order to ensure reliability of interpretation, a second researcher reviewed a purposive sample of 10 articles representing discussions about organ donation and the law change, from a variety of sources and with markedly different headlines and content, and were initially coded as positive, negative or neutral, to check for agreement and consistency in the approach to coding. There was 100% agreement between researchers about the tone of articles and the emerging themes, demonstrating strong inter-rater reliability. The number of positive, negative and neutral articles and comments were then summarised and analysed to determine any patterns such as: changes in tone over time; differences between the tone of articles and that of reader-generated comments; and differences between the tone of reporting in different publications.

##### Assessing article influence

In order to account for the different influence of diverse publications on the public, two measures were used. First, the annual number of views for each news website was obtained from Similarweb to determine how many times each website was visited over the last year. Second, an engagement score was obtained from Alexa Analytics, which is defined as total engagement (number of Twitter retweets, Twitter replies, Twitter likes, Reddit comments and Reddit votes) divided by the total number of articles published (25, 26). This can help to determine which sites have a highly engaged audience who are sharing or engaging with content. Both measures have limitations: the annual number of views includes views from people outside the UK, and is a measure of total views, rather than unique visitors. The engagement score does not include engagement *via* social media sites such as Facebook, or Instagram, and does not include comments directly posted to news websites. Both measures were therefore used independently to provide separate adjustments for influence in the summative content analysis.

##### Thematic analysis

At the end of the summative content analysis, the researcher was familiar with the entire dataset of 286 articles and their content. This familiarity enabled notes and memos to be made on emerging patterns and themes across the entire dataset. This information was then used to support the generation of initial codes and themes for a more detailed thematic analysis of the 21 articles with associated reader-generated comments. These 21 articles with relevant reader-generated comments were read again and where appropriate annotated and coded using NVivo. Additional codes were added as the inductive analysis progressed (Appendix 3). Codes were subsequently grouped and combined to create broader themes, reflecting patterns and ideas within the data. NVivo software automatically grouped together extracts of media articles with the same code, and a thematic map (Appendix 4) was created in order to visualise this. Themes were further defined and refined and complemented by coding from the previously undertaken content analysis of all 286 articles to understand the key ideas which underpinned them. Analysis was undertaken for each individual theme to identify what was being communicated by the news media and reader-generated content. Names for each theme were then finalised and articles were reviewed again to identify appropriate extracts for inclusion.

##### Integrating themes with key findings from the summative content analysis

Where relevant, we juxtaposed relevant key findings from the summative content analysis alongside the thematic analysis to develop an overall interpretation.

### Reflexivity, reliability, and rigour

The first author had no prior experience of research on organ donation but had expertise in public health and mixed-methods analysis. The other five co-authors had expertise in organ donation, health systems and services, public health and policy research and qualitative data analysis, thus bringing different perspectives to the analysis. Co-authors made transparent their positioning and any potential biases. A protocol was developed that included a high level of systematic processing. Decisions were discussed and agreed. Data and emerging findings were shared and discussed among the authors and presented to patient and public representatives who provided additional perspectives and input into the analysis.

## Findings

### Summative content analysis: Media stories

Of the 286 articles analysed, 243 (85%) had a positive tone in relation to organ donation, 25 (9%) were neutral, and 18 (6%) were negative. When articles were weighted based on the annual number of online views for the publication or based on engagement score, the proportion with a positive tone increased (Table 1).

**Table 1:** Summary of content analysis of all media articles and reader-generated comments.

**Tone**	**Organ donation**	**After adjustment for annual views**	**After adjustment for engagement score**
**Summary of content analysis of all media articles**			
Positive	85%	91%	88%
Neutral	9%	4%	6%
Negative	6%	5%	6%
	**Law change**		
Positive	76%	84%	79%
Neutral	19%	12%	14%
Negative	5%	4%	7%
**Summary of content analysis of all reader-generated comments**			
Positive	61%	57%	61%
Neutral	3%	3%	3%
Negative	36%	40%	36%
	**Law change**		
Positive	34%	27%	33%
Neutral	1%	1%	1%
Negative	65%	72%	66%

One hundred and fifty-five (54%) of the 286 articles mentioned the law change to a soft opt-out system. Of these 119 (76%) had a positive tone in relation to the change in law, 29 (19%) were neutral, and 7 (5%) were negative. When articles were weighted according to online views, or according to engagement score, the proportion with a positive tone also increased (Table 1). Further detail of all media articles identified and their tone is available in Appendix 2.

**Figure 2:**
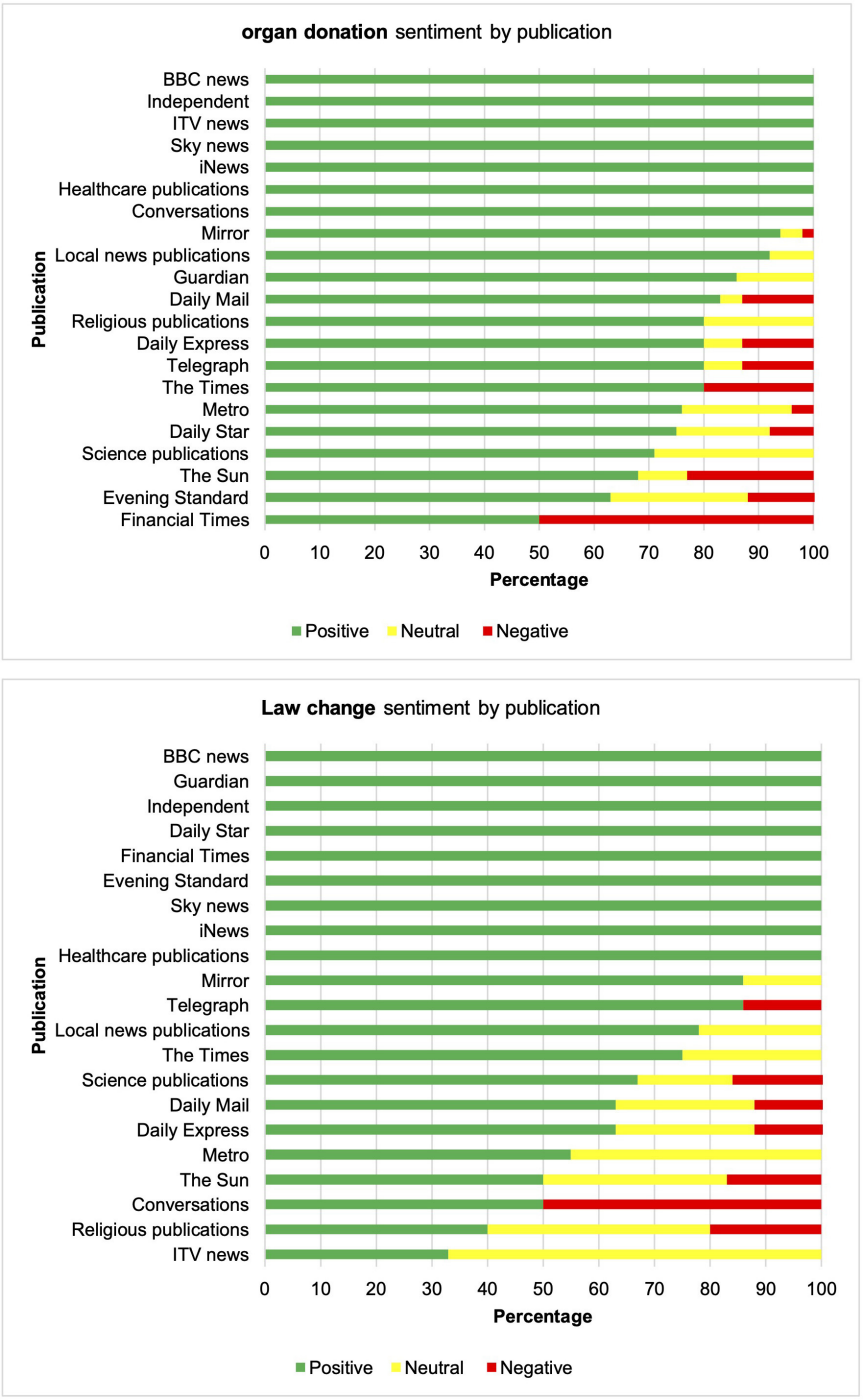
Organ donation and law change sentiment by publication.

**Figure 3:**
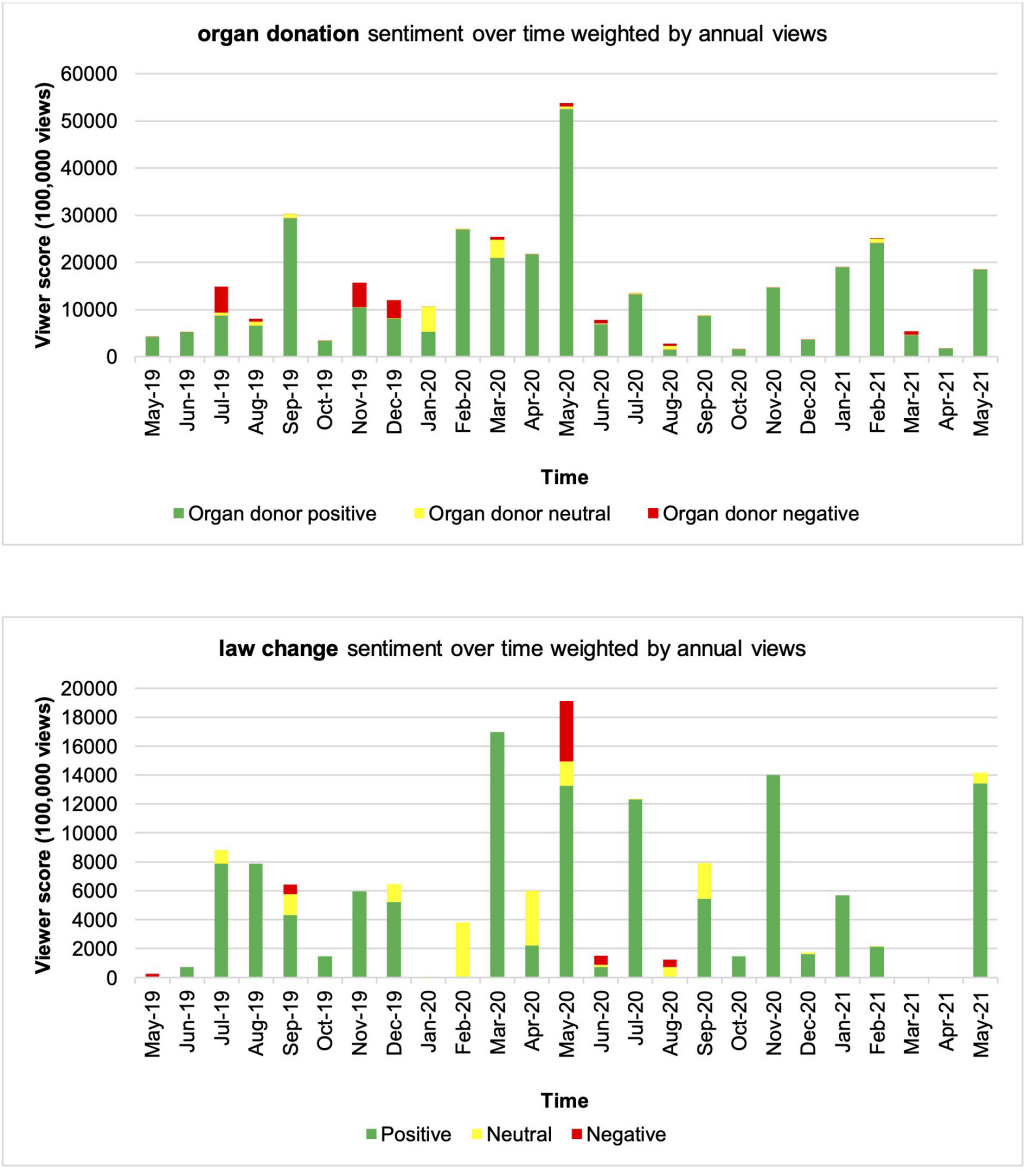
Organ donation and law change sentiment over time weighted by annual views.

A comparison of the tone of articles by publication found some variation, particularly in relation to the law change. All articles published by BBC news, The Guardian, The Independent, Daily Star, Financial Times, Evening Standard, Sky News, iNews and healthcare-related publications conveyed a positive tone in relation to the law change, compared to less than half of articles by religious publications and ITV news. Figure 2 shows variation in the tone of articles by publication. The proportion of positive, neutral and negative stories remained fairly consistent, both in relation to organ donation and in relation to the law change over the period of analysis (20 May 2019 to 20 May 2021). Figure 3 shows the variation in the quantity and tone of articles over time.

### Summative content analysis: Reader-generated comments

**Figure 4:**
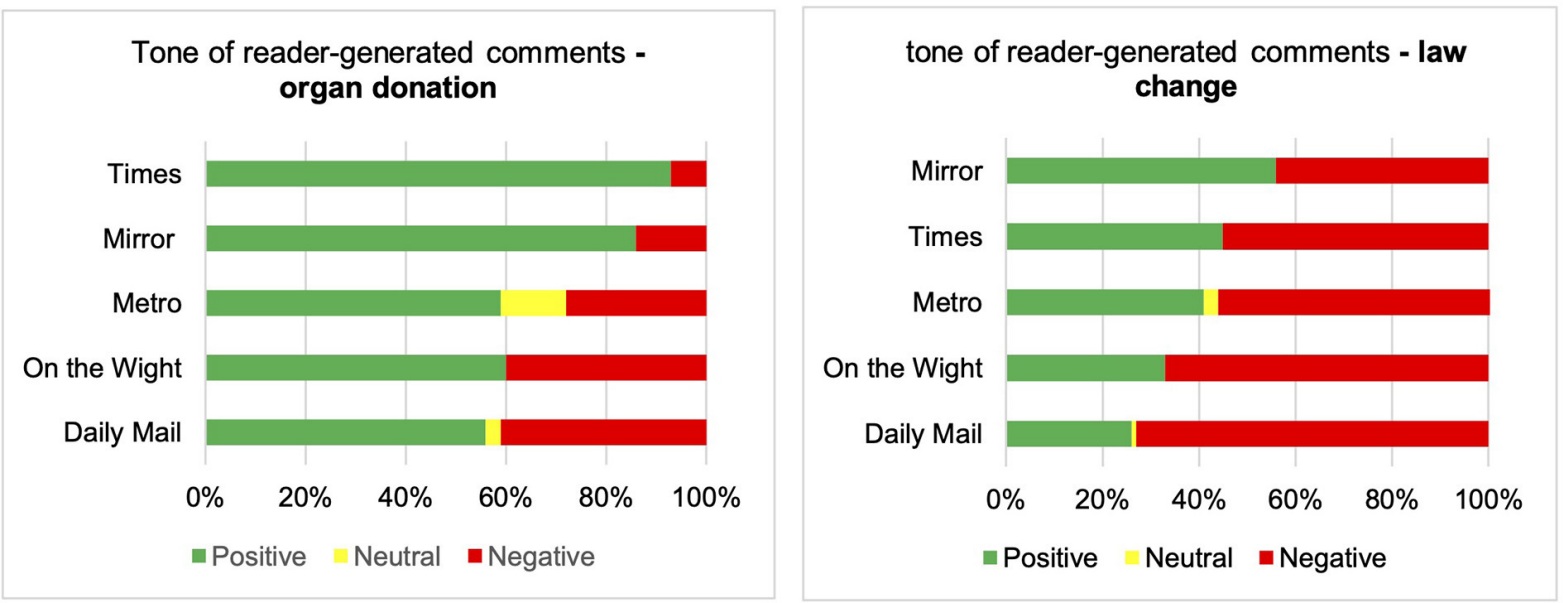
Tone of reader-generated comments—organ donation and law change.

Twenty-one of the 286 articles were accompanied by relevant reader-generated comments. In relation to organ donation, 189 (61%) comments had a positive tone, 10 (3%) were neutral while 110 (36%) were negative (Figure 4). These proportions were unchanged when adjusting for engagement score. When comments were adjusted for average number of views per publication, 57% had a positive tone in relation to organ donation, 3% were neutral, and 40% were negative. Three hundred and twenty-one of the relevant reader-generated comments mentioned or referred to the law change. Of these, 109 (34%) had a positive tone in relation to the law change, 3 (1%) were neutral, and 209 (65%) were negative. When comments were adjusted for annual views per publication and engagement score, the proportion with a positive tone fell, suggesting comments accompanying the most influential publications were more negative (Table 1). Further details of all reader-generated comments identified and their tone is available in Appendix 2.

The proportion of positive and negative comments varied depending on the publication. Of comments published by the Times, 93% were positive in relation to organ donation, and 7% were negative, while only 56% of comments published by the Daily Mail were positive in relation to organ donation, and 41% were negative. Similarly, of comments published in relation to the change in law, 56% were positive and 44% were negative in The Mirror, while in the Daily Mail, only 26% were positive and 73% were negative (Table 2).

**Table 2:** Content analysis of reader-generated comments by publication.

	**Daily Mail**	**Metro**	**On the Wight**	**The Times**	**The Mirror**	**Total**
**Organ donation tone**						
Positive	56%	59%	60%	93%	86%	61%
Neutral	3%	13%	0%	0%	0%	3%
Negative	41%	28%	40%	7%	14%	36%
**Law change tone**						
Positive	26%	41%	33%	45%	56%	34%
Neutral	1%	2%	0%	0%	0%	1%
Negative	73%	57%	67%	55%	44%	65%

### Thematic analysis (complemented by coded data from the content analysis)

Six themes were developed from the data:

1. The importance of organ donation for recipients and donor families
2. Inequalities
3. The quality of organs which become available
4. An NHS under pressure
5. “Scientists playing God”
6. Tensions between the rights of individuals and those of the state.

### Theme 1. The importance of organ donation for recipients and donor families

The majority of media stories emphasised the importance of deceased organ donation, both for recipients and for families of donors. At least 115/286 (40%) of articles featured personal stories about people who were waiting for, or who had received, an organ transplant. These stories were typically highly emotive. They almost exclusively featured babies, children, young people or parents of young children. Media stories typically emphasised the rare or unusual circumstances of both donors and recipients, describing the death of a donor as a “freak” or “tragic” accident (The Sun, 27 July 2019), while potential recipients were described as suffering from “rare” and often “genetic” conditions (The Mirror, 30 Dec 2019). In addition, emotive language was inserted throughout the stories, describing circumstances as “heart breaking” (Guardian, 29 June 2019), and families waiting for organs as “desperate” (Mirror, 14 Sep 2019). Organ donation was also frequently described as a “miracle” (Manchester Evening News, 04 June 2020), while organ donors and their families were depicted as “brave,” “selfless,” and “heroic” (The Guardian, 13 Nov 2019).

#### Reader-generated comments in response

Reader-generated comments on the whole supported the sentiments described in the media stories but there was more emphasis on, and preference for, choice in terms of who organs were donated to, as seen in this comment:

“I am happy for them to have mine but…I would like a say in who has them. I would be against say [my] liver going to an alcoholic or lungs going to a smoker and would rather somebody with an illness which isn't self-inflicted gets them.” (Disillusioned me, Daily Mail, 2020).

#### Integrated analysis of this theme

These stories emphasised the potential for organ donation to save and improve quality of life, and to provide pride and comfort for the families of deceased relatives. By focusing on children and young people, as well as those with rare or genetic conditions, such articles could inadvertently create a dichotomy between those who are considered to “deserve” an organ (children, suffering from a rare genetic condition that cannot be prevented), and those who do not (older people with chronic conditions, which may be exacerbated by behaviours such as drinking alcohol or smoking).

In addition, by characterising those who donate organs as “superheroes,” and the process of organ donation itself as a “miracle,” organ donors and organ donation were presented as exceptional or unusual and often glamorised.

While emphasising the benefits of organ donation for both donor families and recipients is helpful in framing organ donation as a positive act, the volume of media stories which emphasised the extraordinary qualities of both donor families and recipients did not necessarily contribute to normalising organ donation and may support a more divisive narrative framed in terms of “deserving” and “undeserving” recipients of organs.

### Theme 2. Inequalities

38/286 (13%) articles mentioned the disparity in rates of organ donation between different ethnic groups, highlighting both that people from minority ethnic communities are more likely to need an organ, and that there are fewer minority ethnic organ donors, resulting in poorer outcomes for people from minority ethnic communities. The impact of social and cultural norms was highlighted as a key contributor to this.

“Ashley Asomani, 39, known as Ace, the BBC Radio 1 XTRA DJ, is waiting for a kidney and said that his mother, who is of Ghanian heritage, told him as a teenager “we don't do” organ donation… He said the topic was “really taboo” in his family.” (The Times, 11 Sep 2020).

Religion was also cited as a factor contributing to inequalities by several media publications, although the Metro (13 Sep 2020), Daily Mail (02 Jan 2021) and Religion News (10 Feb 2020), attempted to counter the narrative that religious beliefs were a barrier to organ donation by providing examples of religions or religious leaders that permitted and endorsed organ donation.

“On our website you can see references from well-respected Islamic scholars from Egypt to Singapore, and from USA to the Netherlands, all of which say that organ donation in Islam is allowed. In 2019 a fatwa was even written in the UK by an Imam with 20+ years as a hospital chaplain, which states that organ donation is permissible.” (Metro, 13 Sep 2020),

“Some believe, wrongly, that their religion expressly forbids organ donation.” (Daily Mail, 02 Jan 2021),

“For the Sikh community, the concept of seva or selfless service is a fundamental principle of the faith… our fundamental faith encourages us to spend all our life becoming detached from our body… in our faith, once you've left your body, it's just an empty vessel that's going to decay in the ground or be cremated.” (Religion News, 10 Feb 2020).

Similarly, the Jewish Chronicle ran with a headline in May 2020 that the *Chief Rabbi backs new organ donation system in England*, and many news stories over the period emphasised that religious beliefs would be taken into account when decisions about organ donations were made.

#### Reader-generated comments in response

The reader-generated comments that accompanied such articles sometimes directed blame towards minority ethnic communities for longer waits and poorer outcomes, dismissed the concerns of minority ethnic groups and individuals as “superstitious,” and emphasised the concept of reciprocity:

“It has been understood that the [Black, Asian and Minority Ethnic] BAME do not act as donors generally. This is possibly due to religion or other superstition, unfortunately.” (Bolter, Daily Mail, 2020),

“Things should change within the BAME community. If anyone is willing to accept an organ then they must be willing to donate theirs; or not accept a donation, ever.” (Overlaxed, Daily Mail, 2020).

Media responses to the shortage of minority ethnic donors, and reader-generated comments also highlighted misinformation about organ donation and lack of trust in information, in the government, and in the healthcare system both in the UK and supposed country of origin. Structural racism was described as an important factor in eroding trust and generating fear, which in turn appeared to contribute to a greater proportion of people from minority ethnic communities deciding to opt-out and declining deceased organ donation:

“Misinformation abounds and individuals from communities with reason to feel vulnerable enough already from unconscious bias within healthcare and institutional racism feel they have to take extraordinary measures to protect themselves.” (Ekd, The Times, 2020),

“Older South Asians often have hesitations and questions about the prospect of donation: Will the nurses look after me if I'm ill and dying, or will they just be interested in getting my organs?… Many immigrants from India, familiar with corruption in the medical system and stories of organ donation and trafficking there, also fear organ donation in Britain.” (Religion news, 2020).

#### Integrated analysis of this theme

The volume and nature of the coverage in relation to inequalities and organ donation helped raise the profile of the gap in organ transplant availability and health outcomes and acted as a call to action for people from minority ethnic communities to discuss organ donation and consider donating their organs. The coverage was wide-ranging and mostly nuanced, emphasising not only the impact of religious beliefs and cultural norms, but also the role of misinformation, lack of trust and systemic racism, although some media stories and comments potentially exacerbated the situation by blaming minority ethnic communities and dismissing their concerns.

### Theme 3. The quality of organs which become available

32/286 (11%) of media stories referenced the impact of age or health conditions (including obesity and COVID-19) on the quality of available organs. In most instances, these issues were mentioned briefly and sensitively, and were discussed as part of a wider picture of organ donation and transplant rates. However, there were some notable examples where the quality of available organs was presented in a more sensationalist way, such as:

*People dying fatter and older is “reducing the numbers of useable donated organs” as NHS reveals one in SIX body parts now get rejected by doctors* (Daily Mail, 18 July 2019),

*New figures reveal one in five organ transplants come from drug users* (Daily Mail and Daily Express, 07th & 10th July 2019),

*Woman died of HIV from donor's kidney* (The Times, 10 July 2019),

*Woman dies after receiving “double lung transplant from donor with Covid-19,” report finds* (Evening Standard, 24 Feb 2021),

*One NHS patient died and another left seriously ill after receiving infected donor organs* (The Sun, 21 Nov 2019).

#### Reader-generated comments in response

Comments in responses to stories around the quality of organs were frequently indignant.

“Well pardon me for not being healthy enough for you to harvest my body.”(Fourfifteen, Daily Mail, 2019).

In response to the headline that organs frequently come from drug users, some felt that by transplanting organs perceived to be lower quality, clinical best practise and decision making were abandoned in favour of not wasting organs, time and resources.

#### Integrated analysis of this theme

These headlines spread concern among the public as to the quality of some of the organs being transplanted, and suggests that organs are sometimes coming from people with “bad” habits (e.g., obesity, drug users).

### Theme 4. An NHS under pressure

The overwhelming wave of public support for the NHS during the period of analysis due to COVID-19 meant that media coverage relating to organ donation focused primarily on the pressure the NHS was under and the cancellation of organ transplant operations. 23/286(8%) articles mentioned the drop in number of transplants taking place.

*Exclusive: NHS trusts suspend life-saving organ transplants* (Health Service Journal, 02 April 2020),

*NHS bosses admit ALL organ transplants could be scrapped “within days” over fears patients will catch coronavirus as outbreak overwhelms intensive care units* (Daily Mail, 03 April 2020),

*Coronavirus pressures “put organ transplants at risk”* (BBC news, 09 April 2020).

#### Reader-generated comments in response

Commenters reacted with anger and frustration, both towards the NHS itself, “shame on the NHS” (Daily Mail, 03 April 2020), and towards the government for not providing adequate funding, “this whole crisis just shows how terribly underfunded and uncoordinated the NHS is.” (Daily Mail, 03 April 2020).

#### Integrated analysis of this theme

The media created a picture of a health service unable to cope with demand, failing to meet the needs of people who were sick and collapsing while those in power sat back and watched it happen. The initial support for the NHS quickly turned to annoyance and even resentment that despite the sacrifices people were making or had made, COVID-19 did not go away, and the NHS was still in crisis.

### Theme 5. “Scientists playing god”

Twenty-one (7%) of the 286 articles described technological advances in the field of organ donation. Around half of these advances were described in a positive way, using terms such as major breakthrough”:

*Scientists develop a machine that can keep a donated human liver alive for a week outside the body, (Daily Mail, 13 Jan 2020)*.

However, the others developed different narratives and used graphic images and terms such as “mutant” and “science fiction”:

*Plot to create human-animal hybrids using controversial gene editing science approved* (The Sun, 30 July 2019),

*World's first human head transplant could happen in the next 10 years* (Daily Mail, 20 Dec 2019).

#### Reader-generated comments in response

Although some recognised the potential of research to save and improve lives,

“a real breakthrough that should save many more patients” (Dave444, Daily Mail, 2020),

Other comments reflected the sentiments in the articles with multiple references to “Frankenstein” and “scientists playing God.”

“Frankenstein will become a reality” (Farmergeorge, Daily Mail, 2019),

“Death is traumatic. Grief never goes away, we just learn to cope with it. Yet it is nature, it happens to everyone. Our time is different for everyone, but allowing nature to take its course is better than inserting organs from dead people and playing God. What if the child you save then goes on to suffer horrific events afterwards, that death would have spared them from experiencing, who is to blame then?” (LizJ, Daily Mail, 2021).

Although some interpreted developments in research as progressive others saw such technological innovations as being unnatural and unwelcome. Others felt that novel technologies were ultimately dehumanising by taking away the natural order of death and dying.

### Theme 6. Tensions between the rights of individuals and those of the state

A few media stories explicitly referenced tension between the rights of individuals and those of the state, but the rights and responsibilities of individuals and the government represented a significant source of discussion and contention within the reader-generated comments.

#### Reader-generated comments in response to this theme

One hundred and sixty-five comments (over 50% of those that referred to the change in the law) remarked on one or more of four aspects of the tension between the individual and the state. They were:

a. Objection to the change from opt-in to opt-out on principle,
b. Concern that the law change diminishes the altruistic aspect of organ donation,
c. A lack of trust in the state and questioning of government motivations,
d. Understanding and accepting of criteria for brain death and associated terminology.

##### Objection to the change from opt-in to opt-out on principle

One article, published by the Daily Mail in February 2020, *With a new opt-out donation law weeks away, Dr. Martin Scurr and Dr. Max Pemberton question if the NHS should have the right to take our organs*, put forward two different perspectives on the law change, one of which said that:

“The scheme runs the risk of removing organs from those who did not want this to happen but had not registered their objection. This would seriously damage public confidence, and also represents further state intrusion into our lives.” (Daily Mail, 17 Feb 2020).

These sentiments were echoed by a number of comments made in response to stories across all publications, with many commenters declaring that they would now opt-out “on principle” as a result of the change, having previously opted-in:

“I was on the organ donor register right until “deemed consent” came in. Now I have removed myself. I cannot think of anything more unacceptable than “deemed consent.” The state does not own me.” (LibertarianVoice, Mirror, 2020),

“Having in the past registered as a potential donor, I shall now register as a refusenik. The NHS has no right to assume it owns my body parts.” (Mary Rathke, The Times, 2020).

##### Concern that the law change diminishes the altruistic aspect of organ donation

The change in the nature of organ donation from a “gift” to an “expectation” was also widely discussed by readers, even though this was rarely mentioned by the media. Most mainstream media continued to conceptualise organ donors as “heroes” and “selfless,” and the process of organ donation as the “ultimate gift of life” throughout this period, reflecting the key messages in NHSBT's ‘Pass it on' media campaign. One exception to this was an article by Vatican News, published in May 2020, which stated that the Lead Bishop for Healthcare in England supported organ donation in general but did not support the change in the law because,

“it is important that there is a sense of the gift and there can be a sense of intrusion of the state taking over what should primarily be a gift from one person to another.” (Vatican News, 23 May 2020).

Other comments included:

“For decades I carried a donor card. I always thought it should remain a gift after I have gone. Now it is no longer a gift but a demand. I withdrew my consent weeks before the deadline.” (AnonymousMe, Daily Mail, 2020),

“A donated organ is a gift, not a right.” (Crazywitchlady, Daily Mail, 2020).

##### A lack of trust in the state and questioning of government motivations

Another common concern, also not mentioned in the media, was the suggestion that the law change on organ donation was the start of a “slippery slope” to further state control, and towards the creation of a totalitarian or dystopian society focussed on financial incentives and social cleansing.

“When the state effectively takes ownership of our bodies then we should be worried. I am a donor already but this is going too far.” (Michael Organ, Metro, 2020),

“Sinister legislation. Keep your hands off my intestines.” (Maximus Glutimus, Daily Mail, 2020),

“Turning every human being into spare body parts for others isn't progress however well-intentioned—it's positively chilling… The timing is cynical. Our collective attention is elsewhere. We need to push back against this Orwellian development.” (Bailey, The Times, 2020),

“Look good? Oops, didn't recover after all. Then there are the religions/cultures that put little or no value on females, will there be ‘accidental' deaths of mothers/sisters if a valued son needs an organ transplant? The law is Orwellian and open to abuse and temptation… in so many ways.”(Dorsetmaid, Daily Mail, 2020),

“50,000 to save the life of someone hurt in an accident or 100,000 or more profit made in selling organs to the highest bidder. Schemes started with altruistic intentions have a habit of being hijacked by money-making schemers. The rich will live and the poor will supply parts to keep them going.” (Daily Mail, 2020).

##### Understanding and accepting of criteria for brain death and associated terminology

The view of both the government, and the process of organ donation as something sinister which cannot be trusted, was given further weight by a media article which described a situation in which a teenager who was “certified dead,” “began breathing” again after family consent had been given for organ donation (Mirror, 30 March 2021). Further examples of similar occurrences were echoed by commenters, which highlighted a perceived challenge in accurately defining death and contributed to an overall vision of a government which could not be trusted to prioritise saving lives over “harvesting” organs:

“Friend of mine died recently aged 72. When he was 17 he was involved in a horrendous road accident and was in a coma for over a year. His parents were told he was brain dead and should switch off life support and allow certain organs for transplant. He survived, worked as an accountant, married and raised a family. Brain dead? How many times will this happen?” (Jolleyman, Daily Mail, 2020),

“A person's organs are of no use when their body is completely dead. I think the criterion is brain death which is a condition that cannot always be ascertained with absolute certainty. Several studies have consistently shown the physician's lack of ability to accurately discuss, define and recognise brain death.” (I am David, The Times, 2020),

“OK, as long as the definition of ‘death' doesn't subsequently get revised for the purpose of ensuring the harvested organs are just that little bit fresher.” (Alexander More, The Times, 2020).

#### Integrated analysis of this theme

Implicit in this theme is the idea that the law change was undemocratic and that the timing of its implementation during the COVID-19 pandemic was problematic, as individuals had not had an adequate opportunity to opt-out and make their wishes known. These comments also suggested a lack of trust in the government, with words such as “sinister,” “chilling” and “cynical” hinting that the government must have had an ulterior motive for introducing the legislation, such as to sell organs for money or to save the lives of those members of society considered to be most ‘valuable.'

The use of the word “harvested” is known to be dehumanising, with connotations of farming and cultivating individual organs deliberately for donation. The word “harvest” is rarely used in connection with organ donation by the mainstream media but was mentioned in 69 comments. These differences in use of language reflect further differences in the tone, subject matter and concerns between those published by the media and those written ‘below the line' by readers. While debate about the role of the state, fears about loss of rights, questions about government motivation and loss of trust in the government and NHS were rarely mentioned by the news media, such issues were dominant within the associated reader-generated comments.

## Discussion

The role of news media in shaping as well as reflecting public opinion is well-established. Human interest stories, news about policy changes and new technologies, and opinion pieces from a wide spectrum of publications can influence the way politicians, policymakers and the public view and understand organ donation. This in turn can create and propagate social norms which encourage individuals and families to consent (or not) to deceased organ donation. These analyses show that media messaging on organ donation and the change in the law from May 2019 to May 2021 was largely positive, and that this was consistent across different kinds of publications and over time. Positive stories about the importance of organ donation, accurate reporting of the change in law and its benefits, frequent references to widespread public support for organ donation, emotive human-interest stories, and relatively nuanced reporting of the importance of increasing rates of organ donation in ethnic minority communities created a dominant narrative. This narrative suggested that deceased organ donation is a moral good, as are any measures that help to increase organ donation rates, including the change in the law.

However, in order to increase deceased organ donation rates, the change in the law needed not only to be successfully communicated and supported by the media, but also correctly understood and supported by the public. An important part of this is the changed role of the family to one that supports organ donation decisions rather than makes them. Reader-generated content in the form of online comments ‘below the line' of news articles was different from the articles themselves: attitudes towards organ donation were mixed and those in response to the change in the law were largely negative. Concerns about the expanding role of the state, loss of individual freedoms and rights, the potential for the change in the law to be abused for financial gain, and uncertainty about how death is defined and verified created a counter-narrative to that expressed by the mainstream news media. This narrative suggested that neither the government, nor the NHS could be trusted to act in the best interests of individual patients.

### Meaning of this study in relation to other studies

The majority of coverage by news media in relation to both organ donation and the change in the law was positive, especially when adjusted for the annual number of views, with little change during the period of analysis. This differs from the tone towards the Welsh opt-out policy observed in a similar analysis carried out in 2015–2017 which found that the tone of coverage became more supportive of the Welsh policy over time (12). This difference may be explained, at least in part, by changes in attitudes towards the ‘soft opt-out' law during the intervening period. The introduction of the policy in Wales may have contributed to a positive discourse on the law change, and partial normalisation of the ‘soft opt-out' policy in media reporting across the UK, which may have been further enhanced when early successes from the policy in Wales were reported (27). The move towards more positive attitudes to organ donation is likely to have been supported by The Mirror's ‘Change the Law for Life' campaign, which championed the benefits of an opt-out law for several years, telling the stories of two children, Keira and Max, and presenting a positive view of organ donation. This particular campaign also indicates that the overall intention of the media was to focus on the benefits of organ donation rather than trying to explain or promote a change to a soft opt-out system of organ donation as the law change does not include children.

The carefully crafted NHSBT communications campaign worked in that the majority of articles communicated the law change accurately, with similar content found across multiple publications, and frequently reiterated content from NHSBT's ‘Pass it on' campaign, suggesting a strong relationship between official sources including the NHS and media reporting.

However, while content and messages were relatively consistent across media articles, the tone of reader-generated comments were markedly different: only 27% of comments had a positive tone in relation to the law change, after adjustment for annual views of the publication. This pattern was sustained across publications and was observed even when comments were responding to positive articles. The disparity between the tone of articles and reader-generated comments may be in part a reaction against the overwhelmingly positive coverage of the change to the law in the mainstream media, which may foster a view that the media is acting as the mouthpiece of the state and provoke a negative reaction among commenters. In addition, there is evidence that people are more likely to comment on content they disagree with, and so positive coverage may have been more likely to incite a negative response (28). However, the presence of such comments is likely to alter the perception of other readers about public opinion of the law change and may also affect perception of the news itself (29). This may in turn influence wider public views and thus undermine the aims of both NHSBT and the mainstream media in normalising organ donation following the introduction of the soft opt-out policy. Recent research by Ferguson et al. found that even those who may initially wish to co-operate with becoming an organ donor by default and feel encouraged by the law change, may also be strongly affected by individuals or “lone wolves” who publicly and vocally declare their decision to opt-out, suggesting the impact of comments which do not support organ donation or the change in law may be significant (30).

### Strengths and limitations

Media stories were identified from a wide range of sources, including national and local newspapers, and specialist publications. The volume and variety of coverage identified provided an opportunity to analyse trends over time, identify differences between publications and gain an overview of the tone and content in relation both to organ donation, and the change in the law. The analysis of reader-generated comments, as well as media stories, also facilitated comparisons between media and reader-generated content, and enabled further analysis of how the law change was understood and supported by the public.

The use of a mixed-methods approach, undertaking a summative content analysis across a wide range of articles and a thematic analysis provided an opportunity to gain a broad perspective on the totality of media coverage, while also exploring the themes, language and framing of individual articles and reader-generated comments in more depth. Taking an inductive approach to thematic analysis allowed themes to emerge, rather than imposing pre-conceived ideas which may have constrained the analysis. The use of a second researcher to verify sentiment and coding of a sample of articles also strengthened the reliability of the findings, while weighting the media articles according to views and engagement ensured the results were largely representative of their likely influence on the population.

While the volume of media articles provided a rich view of media sentiment during the period of analysis, reader-generated comments observed are unlikely to be representative of the general public and cannot be interpreted as a proxy for overall public attitudes towards organ donation or the change in the law. This is because those who post comments in response to articles online are likely to differ systematically from the wider population in a number of important ways. First, people are more likely to comment online if they disagree with the sentiment or content of a news story (28), and as the majority of news coverage was positive, those with negative views are likely to be disproportionately represented ‘below the line.' Second, people posting online comments may hold stronger views than the general population, since they are motivated to respond. Third, people posting comments online may be influenced by the physical distance and relative anonymity of the online environment, leading to some commenters posting deliberately provocative content (which they may or may not completely agree with) or posting multiple comments under different names to deliberately undermine public health messages (31). Fourth, comments analysed were from a limited range of publications, with a significant number from the Daily Mail, whose readership is unlikely to be representative of the overall population. In addition, most articles did not contain any online comments, either because there was no facility to comment or comments had been disabled, it was possible to comment but no one had done so, or online comments had been removed by the publication by the time of the search. Nonetheless overall we did see a tendency for the below the line comments in setting a tone and subsequent narrative which did have the capacity to influence other people.

Finally, the COVID-19 pandemic affected media reporting and public responses during the period of analysis. The final stages of the NHSBT media campaign to inform the public about the change in the law were cancelled and NHSBT worked closely with the media to limit the promotion of organ donation at the height of the pandemic. This is likely to have affected coverage and public awareness of the law change, and limited media stories as well as the public's response to them across mainstream media. For example, stories which included COVID-19 in the narrative and painting a picture of a health system in such disarray were especially likely to stoke fear and anxiety and likely contributed to undermining confidence not just in organ donation, but in the wider NHS health system.

### Implications for policy and practise

We do not yet fully understand the short-term impact of the soft opt out in England on public attitudes towards organ donation. A UK-wide study is currently underway investigating trends in organ donation consent rates, organ utilisation, adaption of opt-out across the health system and experiences of families who were approached about organ donation after their relative died. A part of this wider study is an investigation into the public's attitudes, views and behaviour (e.g., in relation to the organ donor register) following the changes. The findings should help with more nuanced identification of population sub-groups who are less convinced of the merits of the law change and who might be the focus of NHSBT's media campaigns. The current analysis shows that despite carefully crafted positive messaging, divisive narratives and misinformation dominated the reader-generated content during this time, alongside legitimate concerns about the potentially expanded scope of state involvement in decisions about deceased organ donation.

### Recommendations and further research

We were unable to undertake any detailed analysis of comments, threads and discussion posted on social media sites in connection with the media stories included here. We identify this as a gap and an area for future study, namely the impact of social media on interventions designed to increase number of organs available for transplant and to establish the influence of social media posts on wider public sentiment around living and deceased organ donation. Finally we note an overall lack of evidence speaking to the effectiveness of mass organ donation media campaigns and encourage robust evaluations capable of measuring what matters to the multiple stakeholders are built into future communication planning (32).

## Conclusion

The way the public disseminate and consume information has changed rapidly in recent years. Reader-generated comments have an increasing capacity and capability to shape narratives and understanding of media content, even when these discourses are unreconcilable with the source story. Organ donation remains sensitive and poorly understood across large parts of the general public. The views represented in online comments reveal an important alternative viewpoint to those presented by the mainstream media in relation to organ donation and soft opt-out policies designed to increase the number of organs available for transplant. These views are likely to influence the interpretation and understanding of other readers although we do not yet fully understand how these relationships work, their interdependencies, and the full impact on public attitudes and behaviour in relation to organ donation. Additional tailored interventions are needed (e.g., evidence-based community centred approaches, targeted messaging to reflect local reality and social identities and education to minimise communications which may promote fear, avoidance or denial), alongside future media campaigns, to address mis- and dis-information and ensure that the public continue to have access to trustworthy sources and reliable guidance which are likely to vary for different subgroups.

## Data availability statement

The original contributions presented in the study are included in the article/Supplementary material, further inquiries can be directed to the corresponding author/s.

## Author contributions

GF undertook primary data collection and analysis, and drafted the first version of the manuscript. LW supervised data collection, analysis, and drafting the first version of the manuscript. JN supported drafting the research question and study design, provided feedback on data collection and analysis, provided feedback on early versions, and approved the final version of manuscript. LM supported the study design and data analysis, undertook comprehensive edit of manuscript, prepared the manuscript for submission, and approved the final version of the manuscript. JB reviewed the manuscript and provided comments for submission. NM supported drafting the research question and study design, provided feedback on data collection and analysis, provided feedback on the first version, commented in detail on a subsequent version, and approved the final version of the manuscript. All authors contributed to the article and approved the submitted version.

## Funding

This study was part-funded by the NIHR Policy Research Programme through its core support to the Policy Innovation and Evaluation Research Unit (Project No. PR-PRU-1217-20602).

## Conflict of interest

The authors declare that the research was conducted in the absence of any commercial or financial relationships that could be construed as a potential conflict of interest.

## Publisher's note

All claims expressed in this article are solely those of the authors and do not necessarily represent those of their affiliated organizations, or those of the publisher, the editors and the reviewers. Any product that may be evaluated in this article, or claim that may be made by its manufacturer, is not guaranteed or endorsed by the publisher.

## Author disclaimer

The views expressed are those of the author(s) and are not necessarily those of the NIHR or the Department of Health and Social Care.
