# Supplementary material for: Analysis of content and online public responses to media articles that raise awareness of the opt-out system of consent to organ donation in England
Source: Front Public Health. 2022 Dec 1;10:1067635. doi: 10.3389/fpubh.2022.1067635 (PMC9751921; doi:10.3389/fpubh.2022.1067635)
Supplement: Supplementary file 1 [file Table_1.DOCX]

| **Publication** | **Estimated number of readers (2020)** |
| --- | --- |
| Metro | 1,426,535 |
| The Sun | 1,250,634 |
| Daily Mail | 1,169,241 |
| Evening Standard | 798,168 |
| Daily Mirror | 451,466 |
| The Times | 368,929 |
| Daily Telegraph | 360,345 |
| Daily Express | 296,079 |
| Daily Star | 277,237 |
| i | 217,182 |
| Financial Times | 157,982 |
| The Guardian | 132,341 |
| Daily Record | 104,343 |
| City A.M. | 85,521 |
| The Independent | 55,193 |
