# Supplementary material for: Analysis of content and online public responses to media articles that raise awareness of the opt-out system of consent to organ donation in England
Source: Front Public Health. 2022 Dec 1;10:1067635. doi: 10.3389/fpubh.2022.1067635 (PMC9751921; doi:10.3389/fpubh.2022.1067635)
Supplement: Supplementary file 2 [file Table_2.DOCX]

| **Publication** | **Date of publication** | **Headline** | **Organ donation tone** | **Law change tone** | **Viewer score** | **Engagement score** |
| --- | --- | --- | --- | --- | --- | --- |
| iNews | 20 May 2019 | “I’ve just turned 30 – it’s a birthday I never thought I’d see”: Cystic fibrosis sufferer celebrates life after double lung transplant | Positive | Positive | 119 | 57 |
| Evening Standard | 23 May 2019 | Machine that keeps livers ‘alive’ could allow for more life-saving transplants in future | Positive | N/A | 282 | 64 |
| Daily Mail | 28 May 2019 | Mother of teenage organ donor says she ‘got a sense of Ben’ when she met the father-of-two given her dead son’s liver for the first time at the finish line of a walking challenge | Positive | N/A | 3256 | 117 |
| The Conversation | 29 May 2019 | Opt-out organ donation: presume kindness, not consent, to save more lives | Positive | Negative | 248 | 73 |
| East London Lines | 4 June 2019 | New campaign launched to encourage organ donation among BAME communities | Positive | N/A | 1 | 1 |
| The Star | 8 June 2019 | Three organ donors from Sheffield honoured for saving lives in posthumous award ceremony | Positive | Positive | 26 | 25 |
| Northern Echo | 9 June 2019 | Meet the nurse saving lives by encouraging organ donation | Positive | Positive | 37 | 9 |
| Daily Mail | 10 June 2019 | Breakthrough device that can keep donor organs functioning outside the body for 24 hours will revolutionise transplant surgery, experts say | Positive | N/A | 3256 | 117 |
| The Mirror | 24 June 2019 | Max and Keira’s Law: The story of how the Daily Mirror campaigned to change the law on organ donation | Positive | Positive | 906 | 81 |
| The Metro | 26 June 2019 | Boy, 10, died playing football when ‘blood vessel burst in his brain’ | Positive | N/A | 328 | 40 |
| The Sun | 26 June 2019 | ‘LOVED BY ALL’ Football-mad boy, 10, collapses and dies while playing with pals in the park after suffering cardiac arrest | Positive | N/A | 1069 | 48 |
| Windsford and Middlewich Guardian | 29 June 2019 | “Losing her was such a heartbreaking time but we are so glad we agreed to organ donation” | Positive | Positive | 1 | 2 |
| Leigh Journal | 3 July 2019 | Sisters of 19-year-old who became a life-saving organ donor after his sudden death ‘honoured’ to accept award in his name | Positive | N/A | 1 | 1 |
| Daily Express | 7 July 2019 | New figures reveal one in five organ transplants come from drug users | Negative | N/A | 1351 | 50 |
| Daily Mail | 10 July 2019 | Revealed: one in five organs used in transplants including kidneys, lungs and hearts have come from drug users | Negative | N/A | 3256 | 117 |
| The Times | 10 July 2019 | Woman died of HIV from donor’s kidney | Negative | N/A | 357 | 117 |
| The Mirror | 17 July 2019 | Britain’s longest surviving heart transplant patient makes plea for organ donors | Positive | Positive | 906 | 71 |
| Daily Mail | 18 July 2019 | People dying fatter and older is ‘reducing the number of usable donated organs’ as NHS reveals one in SIX body parts now get rejected by doctors | Positive | Positive | 3256 | 117 |
| The Independent | 18 July 2019 | Obesity and old age blamed as organ transplants fall despite record number of donors | Positive | Positive | 937 | 287 |
| The Telegraph | 18 July 2019 | Soaring obesity rates fuel doubling in the number of organs which cannot be transplanted | Neutral | Positive | 609 | 107 |
| Finchley and Barnet Times | 19 July 2019 | Patient visits Barnet school over change in law for organ donations | Positive | Neutral | 1 | 9 |
| The Metro | 19 July 2019 | Teen saves lives after ‘mysteriously’ becoming organ donor two weeks before dying | Positive | N/A | 328 | 40 |
| Essex Gazette | 22 July 2019 | Only 25 people eligible for organ donation in Essex | Positive | Neutral | 13 | 4 |
| Daily Express | 26 July 2019 | British Transplant Games poignant for Wilson family as Tom’s memory lives on | Positive | N/A | 135 | 50 |
| The Metro | 26 July 2019 | The reality of having an organ transplant is not what you think | Positive | Neutral | 328 | 40 |
| London news | 26 July 2019 | Organ donations from St George’s Hospital helps the UK reach its highest level of donors | Positive | Positive | 6 | 19 |
| The Sun | 27 July 2019 | FAMILY TORN APART Heartbroken mum loses son, 22, in freak hockey accident and husband, 56, from sepsis just weeks apart | Positive | N/A | 1069 | 48 |
| Essex Live | 27 July 2019 | Essex mum whose son and husband died eight weeks apart urges people to donate their organs | Positive | N/A | 27 | 8 |
| Lancs Live | 29 July 2019 | Posthumous honour for organ donor Pippa Astbury who helped save three lives | Positive | N/A | 22 | 10 |
| The Sun | 30 July 2019 | MUTANT MADNESS Plot to create ‘human-animal hybrids’ using controversial gene-editing science approved | Negative | N/A | 1069 | 48 |
| Financial Times | 2 Aug 2019 | Richard Thaler: “If you want people to do something, make it easy” | Neutral | N/A | 268 | 45 |
| Stone and Eccleshall Gazette | 2 Aug 2019 | Call for families in Staffordshire to talk about organ donation | Positive | Positive | 1 | 1 |
| Daily Express | 10 Aug 2019 | George Shelley sister: The tragic accident that killed his sister – but saved five lives | Positive | N/A | 135 | 50 |
| iNews | 17 Aug 2019 | Mother who lost her 22-year-old son holds hands with six-year-old girl who was saved by his liver | Positive | N/A | 119 | 57 |
| The Sun | 17 Aug 2019 | ‘HE’S MY HERO’ Girl, 6, praises the “hero” who helped save her life through organ donation after he died of a brain haemorrhage | Positive | N/A | 1069 | 48 |
| The Telegraph | 17 Aug 2019 | Pig hearts ‘could be used in human transplants within three years’ after gene breakthrough | Positive | Positive | 609 | 107 |
| The Sun | 18 Aug 2019 | FRANKENSWINE Pig hearts could be used in human transplants ‘within just three YEARS’ says surgeon who performed first ever op 40 years ago | Negative | N/A | 1069 | 48 |
| The Telegraph | 18 Aug 2019 | How Britain’s first heart transplant almost didn’t happen due to medical ‘rivalries and intense hostility’ | Positive | Positive | 609 | 107 |
| The Conversation | 20 Aug 2019 | Organ transplants: why so many people are put off donating | Positive | Positive | 248 | 73 |
| Daily Mail | 21 Aug 2019 | Mother whose son died aged 17 ‘after a sneezing fit triggered a stroke’ meets the man whose life was saved by his donor heart | Positive | N/A | 3256 | 117 |
| Financial Times | 21 Aug 2019 | Pig organs to be used in transplant operations | Negative | N/A | 268 | 45 |
| Daily Express | 23 Aug 2019 | Transplant breakthrough: Pig-to-human heart transplants possible ‘within three years’ | Neutral | N/A | 135 | 50 |
| The Mirror | 2 Sept 2019 | Bereaved families objecting to organ donation is costing hundreds of lives | Positive | Neutral | 906 | 81 |
| The Telegraph | 2 Sept 2019 | “I didn’t want my sister to die for nothing: It just seemed such a waste” | Positive | Positive | 609 | 107 |
| Cambridge Independent | 2 Sept 2019 | Organ Donation Week: families urged to talk about their wishes | Positive | Positive | 2 | 16 |
| ITV news | 3 Sept 2019 | New law means organ donation will be automatic after death: What are the facts? | Positive | Positive | 924 | 120 |
| Romford Recorder | 4 Sept 2019 | Organ Donation Week 2019: Queen’s hospital doctor urges organ donors to make family aware of their wishes | Positive | Positive | 3 | 7 |
| The Mirror | 5 Sept 2019 | Mum who lost 5 relatives to kidney disease gets miracle donor alongside mum and sister | Positive | N/A | 906 | 81 |
| The Guardian | 5 Sept 2019 | Organ donations from BAME community at record high, data shows | Positive | N/A | 3046 | 148 |
| BBC news | 6 Sept 2019 | Hepatitis C-infected kidneys used in organ transplants | Positive | N/A | 7781 | 152 |
| Daily Star | 6 Sept 2019 | Tragic mum of eight-month-old-baby ‘took own life’ weeks before 25^th^ birthday | Positive | N/A | 348 | 16 |
| Shropshire Star | 6 Sept 2019 | Organ Donation Week: “to give the chance of life to someone is the most precious gift” | Positive | Positive | 16 | 13 |
| The Telegraph | 6 Sept 2019 | Dozens more kidneys could be donated after doctors successfully cure transplant organ with hepatitis C | Positive | N/A | 609 | 107 |
| The Mirror | 7 Sept 2019 | Teenager with ‘heart of pure gold’ saves three lives with organ donors | Positive | N/A | 906 | 81 |
| The Sun | 7 Sept 2019 | DEATH SENTENCE Busy mum who went to the doctor with tiredness was told her liver was shrinking and she had 72 hours to live | Neutral | N/A | 1069 | 48 |
| The Sun | 7 Sept 2019 | BRIDE AND JOY Double transplant took diabetes suffering dad, 35, from contemplating last rites to tying the knot | Positive | N/A | 1069 | 48 |
| The Metro | 9 Sept 2019 | Man ‘wanted to die’ after rare illness caused belly to swell up like he was pregnant | Neutral | N/A | 328 | 40 |
| The Mirror | 14 Sept 2019 | Woman ‘slowly dying’ with months to live makes desperate plea for transplant | Positive | Positive | 906 | 81 |
| The Mirror | 20 Sept 2019 | Brave youngster Max Johnson to be celebrated as ‘Heart Hero’ at awards ceremony | Positive | Positive | 906 | 81 |
| The Telegraph | 24 Sept 2019 | Heart transplant waiting lists hit record high, with doubling in number waiting | Positive | Positive | 609 | 107 |
| The Times | 24 Sept 2019 | Longer wait for heart transplants as donor quality drops | Positive | Positive | 357 | 117 |
| Daily Mail | 26 Sept 2019 | Kind hearted teenager’s organs saved three lives after 18-year-old ran into path of oncoming Mercedes and was killed outside her school | Positive | N/A | 3256 | 117 |
| The Metro | 26 Sept 2019 | Schoolgirl ran over and killed ‘after bus driver signalled she could cross road’ | Positive | N/A | 328 | 40 |
| Daily Express | 29 Sept 2019 | Have a heart and save us: Desperate parents plead for child donors | Positive | Negative | 1351 | 50 |
| The Mirror | 29 Sept 2019 | “Stranger’s heart saved my life – now my baby son needs a miracle donor too” | Positive | Neutral | 906 | 81 |
| The Sun | 11 Oct 2019 | School children blow bubbles and mourners dress in blue as ‘hit and run’ death girl Melissa Tate, 10, laid to rest | Positive | N/A | 1069 | 48 |
| Kent Online | 15 Oct 2019 | Tonbridge mum Gemma Ashdown hopes children will hear organ donor dad’s heart beat again | Positive | N/A | 54 | 19 |
| The Mirror | 19 Oct 2019 | Dad who had vital transplant shares ‘special’ bond with organ donor’s parents | Positive | N/A | 906 | 81 |
| The Mirror | 20 Oct 2019 | We put little Max in the public eye to change law – and find him a heart donor | Positive | Positive | 906 | 81 |
| The Metro | 21 Oct 2019 | Woman who survived kidney and liver transplants becomes athletics star | Positive | N/A | 328 | 40 |
| The Mirror | 24 Oct 2019 | Emotional mums who inspired organ donor campaign meet for first time 25 years on | Positive | Positive | 906 | 81 |
| The Sun | 28 Oct 2019 | ‘MOWED DOWN’ Driver admits causing death of ‘beautiful’ girl, 10, in ‘hit-and-run’ | Positive | N/A | 1069 | 48 |
| The Mirror | 3 Nov 2019 | “Our son’s death was not in vain after he gave organs to Team GB winner” | Positive | Positive | 906 | 81 |
| The Mirror | 4 Nov 2019 | Organ donor’s heartbroken wife makes tearful plea to person receiving his heart | Positive | Positive | 906 | 81 |
| The Guardian | 10 Nov 2019 | “I knew my son’s heart was out there”: why the families of organ donors would love a thank you | Positive | Positive | 3046 | 148 |
| The Guardian | 13 Nov 2019 | Overwhelming gratitude for the priceless gift of organ donation | Positive | N/A | 3046 | 148 |
| New Milton Advertiser | 14 Nov 2019 | Accidental death victim saved two lives with organ donation, inquest told | Positive | N/A | 1 | 4 |
| The Telegraph | 15 Nov 2019 | I gave you my heart: the surprising truth behind that Last Christmas organ donation twist | Negative | N/A | 609 | 107 |
| Daily Mail | 21 Nov 2019 | One NHS patient DIED and another became seriously ill after receiving infected organs from surgeon who failed to disclose his fatal mistake | Negative | N/A | 3256 | 117 |
| The Metro | 21 Nov 2019 | Patient died after surgeon spilled stomach contents onto donor’s organs | Negative | N/A | 328 | 40 |
| The Sun | 21 Nov 2019 | SURGICAL BLUNDER One NHS patient died and another left seriously ill after receiving infected donor organs | Negative | N/A | 1069 | 48 |
| Bristol Post | 2 Dec 2019 | The people spreading the word on organ donation to Bristol’s African Caribbean community | Positive | Positive | 40 | 14 |
| The Metro | 3 Dec 2019 | Baby desperate for new heart just months after his dad received a transplant | Positive | N/A | 328 | 40 |
| ITV news | 6 Dec 2019 | Nine-month-old baby desperate for new heart to spend Christmas in hospital | Positive | Neutral | 924 | 120 |
| Daily Express | 8 Dec 2019 | All I want for Christmas is a new heart – child donor appeal | Positive | Positive | 1351 | 50 |
| The Mirror | 16 Dec 2019 | Baby born with half a heart receives life-saving Christmas organ donation | Positive | N/A | 906 | 81 |
| The Mirror | 17 Dec 2019 | Incredible Izzy whose kidneys failed at birth rings ‘transplant bell’ for the first time | Positive | N/A | 906 | 81 |
| Daily Mail | 20 Dec 2019 | Moving TV advert encourages families to talk about organ donation this Christmas before the law changes to make EVERY adult a donor next year | Positive | Positive | 3256 | 117 |
| The Metro | 20 Dec 2019 | NHS urges families to share organ donation wishes before opt-out system hits | Neutral | Neutral | 328 | 40 |
| Daily Mail | 22 Dec 2019 | World’s first human HEAD transplant ‘could happen in next ten years’ due to advances in technology, says ex-NHS neurosurgeon and robotics expert | Negative | N/A | 3256 | 117 |
| The Mirror | 30 Dec 2019 | Girl, 12, saved by miracle heart transplant after nearly dying from rare disease | Positive | Positive | 906 | 81 |
| Daily Star | 31 Dec 2019 | Girl, 12, heaps praise on donor’s family following successful heart transplant | Positive | N/A | 348 | 16 |
| SW Londoner | 8 Jan 2020 | BAME lives to be saved as new organ donation law rolls out | Positive | Positive | 2 | 17 |
| Daily Mail | 13 Jan 2020 | Scientists develop a machine that can keep a donated human liver alive for a WEEK outside the body by pumping fresh blood through the crucial organ | Positive | N/A | 3256 | 117 |
| The Guardian | 13 Jan 2020 | Organ donation: new technique can preserve human livers for a week | Neutral | N/A | 3046 | 148 |
| Jewish Chronicle | 22 Jan 2020 | Survey finds ‘significant confusion’ over organ donation among Jews | Neutral | Neutral | 5 | 1 |
| Evening Standard | 23 Jan 2020 | First ever robotic heart could put an end to transplants, scientists say | Neutral | N/A | 282 | 64 |
| Daily Mirror | 23 Jan 2020 | World’s first totally robotic heart will end need for transplants in 10 years | Neutral | N/A | 906 | 81 |
| Daily Express | 24 Jan 2020 | Iain Dale left in tears as heartbreaking call ends in the most beautiful way | Positive | N/A | 1351 | 50 |
| Hull Daily Mail | 24 Jan 2020 | The organ donation hero who had a miracle year then gifted life after death | Positive | Positive | 47 | 12 |
| Ely Standard | 2 Feb 2020 | Cambridge University Hospitals urge people to become life-saving organ donors | Positive | Positive | 1 | 1 |
| Lexology | 2 Feb 2020 | Give or take? The new law on organ donation | Neutral | Neutral | 30 | 3 |
| BBC news | 7 Feb 2020 | My newborn son – the organ donor | Positive | N/A | 7781 | 152 |
| Religion news | 10 Feb 2020 | Fighting taboos, British Sikhs work to demystify organ donation | Positive | Positive | 11 | 158 |
| Daily Mail | 14 Feb 2020 | Pioneering heart transplant device can keep donated organs alive for 24 HOURS, potentially saving thousands of lives | Positive | N/A | 3256 | 117 |
| Mobile Marketing | 14 Feb 2020 | NHSBT goes social to raise awareness of organ donation law change | Positive | Positive | 1 | 4 |
| The Sun | 14 Feb 2020 | HEART IN A BOX Doctors keep heart beating for 24 hours with new ‘game-changing’ device | Positive | N/A | 1069 | 48 |
| The Telegraph | 14 Feb 2020 | Hearts can be kept alive for 24 hours after death, scientists have shown | Positive | N/A | 609 | 107 |
| Daily Mail | 17 Feb 2020 | With a new opt-out donation law weeks away… DR MARTIN SCURR and DR MAX PEMBERTON question if the NHS should have the right to take our organs? | Positive | Neutral | 3256 | 117 |
| Lancashire Post | 20 Feb 2020 | People across the north west must ensure they get their facts straight about organ donation law change, warn health bosses | Neutral | Neutral | 7 | 7 |
| Leigh Journal | 24 Feb 2020 | Lifts at hospital trust given new look to encourage people to join organ donation register | Neutral | Neutral | 1 | 1 |
| The Mirror | 25 Feb 2020 | Everyone will be an organ donor by default within weeks – thanks to two special children | Positive | Positive | 906 | 81 |
| Nursing Times | 25 Feb 2020 | Exclusive: organ donation nurse network expanded ahead of law change | Positive | Positive | 13 | 79 |
| Sky news | 25 Feb 2020 | Max and Keira’s law: new ‘opt-out’ organ donor system to be introduced on 20 May, government plans | Positive | Positive | 538 | 413 |
| The Independent | 25 Feb 2020 | Adults to be automatically enrolled as organ donors under new law | Positive | Positive | 937 | 287 |
| The Telegraph | 26 Feb 2020 | All adults will be assumed organ donors unless they opt-out under new system | Positive | Positive | 609 | 107 |
| The Guardian | 1 March 2020 | New law on organ donations could save thousands like 12-year-old Max | Positive | Positive | 3 | 148 |
| The Sun | 10 March 2020 | My husband registered to be an organ donor and didn’t tell me… now I don’t trust him again | Negative | N/A | 1069 | 48 |
| Healthcare IT News | 11 March 2020 | Organ donation law seeing move to opt-out system to come into effect in England | Positive | Positive | 5 | 14 |
| The Moorlander | 11 March 2020 | Organ donation law to come into effect | Positive | Positive | 1 | 1 |
| BBC news | 22 March 2020 | Vinnie Jones welcomes organ donation change after wife’s death | Positive | Positive | 7781 | 152 |
| The Metro | 22 March 2020 | Vinnie Jones says wife Tanya’s heart transplant ‘saved his life’ as well | Positive | Positive | 328 | 40 |
| The Sun | 22 March 2020 | “IT GAVE US 32 YEARS” Vinnie Jones says wife’s heart transplant saved HIS life as well as hers – as he praises organ donor law change | Positive | Positive | 1069 | 48 |
| The Metro | 23 March 2020 | Mum listens to late daughter’s heart as it beats in another teenager’s body | Positive | N/A | 328 | 40 |
| Daily Mail | 31 March 2020 | Family of father, 27, who took his own life share their pride at knowing he saved SIX people by donating his organs and say it’s a comfort to know ‘his heart is beating in someone else’ | Positive | N/A | 3256 | 117 |
| Daily Mail | 31 March 2020 | Every organ donor is being tested for coronavirus as NHS rejects transplants from infected patients to minimise risk to recipients | Neutral | N/A | 3256 | 117 |
| The Mirror | 2 April 2020 | Family shed tears for life-saving daughter crowned world’s youngest organ donor | Positive | Positive | 906 | 81 |
| The Mirror | 2 April 2020 | Eddie Large helped change UK organ donation law after receiving heart transplant | Positive | Positive | 906 | 81 |
| Health Service Journal | 2 April 2020 | Exclusive: NHS trusts suspend life-saving organ transplants | Positive | N/A | 3 | 68 |
| Cambridge News | 3 April 2020 | Cambs family shed tears for life-saving baby crowned world’s youngest organ donor | Positive | Positive | 23 | 17 |
| Daily Mail | 3 April 2020 | NHS bosses admit ALL organ transplants could be scrapped ‘within days’ over fears patients will catch coronavirus as outbreak overwhelms intensive care units | Positive | Neutral | 3256 | 117 |
| BBC news | 9 April 2020 | Coronavirus pressures ‘put organ transplants at risk’ | Positive | N/A | 7781 | 152 |
| Daily Mail | 13 April 2020 | Nursery worker, 21, who was waiting for life-saving liver operation dies after being taken off transplant list when she tested positive for coronavirus in hospital | Positive | N/A | 3256 | 117 |
| The Mirror | 27 April 2020 | Boy, 12, saved by 9-year-old donor vows to ‘cherish your gift with all my life’ | Positive | Positive | 906 | 81 |
| EurekAlert | 6 May 2020 | Study reveals impact of ‘soft opt-out’ system for organ donation | Positive | Positive | 22 | 71 |
| BBC news | 12 May 2020 | Coronavirus: low level of transplants sparks concern | Positive | N/A | 7781 | 152 |
| The Mirror | 17 May 2020 | Mum who nearly died after birth saved by miracle heart transplant | Positive | Positive | 906 | 81 |
| The Sun | 17 May 2020 | ‘WORRYING TIME’ Transplant patients face anguish as number of organ donors falls during coronavirus crisis | Positive | N/A | 1069 | 48 |
| The Mirror | 18 May 2020 | Boy, 5, is symbol of hope that new ‘opt out’ transplant system will save lives | Positive | Positive | 906 | 81 |
| iNews | 19 May 2020 | ‘Game changing’ organ donation law means adults in England are deemed to have given consent to donate their organs when they die | Positive | Positive | 119 | 57 |
| The Guardian | 19 May 2020 | All adults in England to be deemed organ donors in ‘opt-out’ system | Positive | Positive | 3046 | 148 |
| The Jewish Chronicle | 19 May 2020 | Chief Rabbi backs new organ donation system in England | Positive | Positive | 5 | 1 |
| The Mirror | 19 May 2020 | Heart transplant hero Max, 12, says “You did it for me… now do it for Ethan” | Positive | Positive | 906 | 81 |
| Daily Express | 20 May 2020 | Organ donation update: every adult now an organ donor unless they opt out | Positive | Positive | 1351 | 50 |
| Daily Express | 20 May 2020 | Lifeline for patients as organ donation by all becomes law | Positive | Positive | 1351 | 50 |
| Evening Standard | 20 May 2020 | New law will see adults in England automatically become organ donors in hope for patients awaiting life-saving transplants | Positive | Positive | 282 | 64 |
| iNews | 20 May 2020 | Organ donation opt-out explained: how to opt out, why the UK law is changing and excluded groups in the NHS scheme | Positive | Positive | 119 | 57 |
| Sky News | 20 May 2020 | England moves to ‘opt out’ organ donation system after change in law | Positive | Positive | 538 | 413 |
| The Independent | 20 May 2020 | Organ donation: how have the laws in England changed and do you still have a choice? | Positive | Positive | 937 | 287 |
| The Metro | 20 May 2020 | How has the law around organ donation changed today and what is the opt-out system? | Positive | Positive | 328 | 40 |
| The Metro | 20 May 2020 | From today every adult in England is automatically an organ donor | Positive | Positive | 328 | 40 |
| The Metro | 20 May 2020 | The new organ donation law will save lives, but it’s pointless if your family don’t know your wishes | Positive | Positive | 328 | 40 |
| The Sun | 20 May 2020 | TICKING TIME BOMB My baby boy needs a new heart… without it, he won’t live to see his 2^nd^ birthday | Positive | N/A | 1069 | 48 |
| The Sun | 20 May 2020 | MAX & KEIRA’S LEGACY Every adult is now an organ donor unless they opt out – thanks to two inspiring children | Positive | Positive | 1069 | 48 |
| The Sun | 20 May 2020 | THE GIFT OF LIFE How does organ donation work and what is Max and Keira’s Law? | Positive | Neutral | 1069 | 48 |
| The Sun | 20 May 2020 | ORGAN DON’TOR How to opt out of organ donation in the UK | Neutral | Neutral | 1069 | 48 |
| The Times | 20 May 2020 | Every adult automatically an organ donor by law | Positive | Positive | 357 | 117 |
| Wired | 20 May 2020 | New organ donation laws could help fix the BAME donor crisis | Positive | Positive | 56 | 128 |
| Daily Mail | 21 May 2020 | I admit it, I feel queasy about the State having a claim on my organs… unless I choose to opt out, writes Melanie McDonagh | Positive | Negative | 3256 | 117 |
| Halifax Courier | 21 May 2020 | Parents of Halifax boy who saved six lives through organ donation support new ‘opt-out’ law | Positive | Positive | 3 | 15 |
| Daily Express | 22 May 2020 | Organ donation opt out: How do I opt out of organ donation scheme? | Negative | Neutral | 1351 | 50 |
| Vatican News | 23 May 2020 | Bishop for Healthcare on England’s new organ donation law | Positive | Negative | 100 | 364 |
| The Mirror | 2 June 2020 | Boy marks first birthday as donor law comes into force after his own transplant | Positive | Positive | 906 | 81 |
| The Mirror | 2 June 2020 | Every member of this choir has been touched by organ donation in some way | Positive | Neutral | 906 | 81 |
| Manchester Evening News | 4 June 2020 | Mum thanks kidney donor who saved her life and allowed her to have her “miracle” baby | Positive | Positive | 346 | 119 |
| Daily Mail | 6 June 2020 | We’re all organ donors now – unless we opt out. Katie Hind’s moving story about her father’s incurable disease and how a new heart saved his life shows why the new law is so essential | Positive | Positive | 3256 | 117 |
| The Telegraph | 12 June 2020 | Letter: a sceptical view of the ‘gods in white’ | Negative | Negative | 609 | 107 |
| Daily Express | 14 June 2020 | Fear Brits died waiting for organ transplants during lockdown as ops plumet by two thirds | Positive | N/A | 1351 | 50 |
| Financial Times | 17 June 2020 | Letter: the patient is not the only transplant beneficiary | Positive | Positive | 268 | 45 |
| Daily Star | 26 June 2020 | Extreme sex killer caged in ‘Monster Mansion’ wants to donate his kidney | Negative | N/A | 348 | 16 |
| The Metro | 28 June 2020 | Boy waiting on organ transplant gets amazing rocket ship bed from grandad to fulfil astronaut dream | Neutral | N/A | 328 | 40 |
| BBC news | 3 July 2020 | Organ donation: “Mum said we don’t do it. So we don’t” | Positive | Positive | 7781 | 152 |
| The Sun | 8 July 2020 | TWO LITTLE MIRACLES My two little girls almost died from deadly heart condition but doctors’ brilliance and strangers’ kindness saved them | Positive | Positive | 1069 | 48 |
| Daily Star | 10 July 2020 | Dad who died after he was ‘punched in Screwfix queue-jumping row’ pictured | Positive | N/A | 348 | 16 |
| EurekAlert | 10 July 2020 | New study warns of misinformation about opt-out organ donation | Positive | Negative | 22 | 71 |
| The Mirror | 16 July 2020 | Our little princess urgently needs new organs – just like other children waiting for call | Positive | N/A | 906 | 81 |
| Daily Star | 20 July 2020 | F1 grid girl in coma after going blind in one eye and suffering kidney failure | Neutral | N/A | 348 | 16 |
| The Leader | 21 July 2020 | Increased public support for organ donation despite impact of Covid-19 | Positive | Positive | 9 | 5 |
| Evening Standard | 22 July 2020 | Kidney transplant resume for patients who have isolated | Neutral | N/A | 282 | 64 |
| Bracknell News | 23 July 2020 | Berkshire families urged to talk about organ donations | Positive | Positive | 4 | 3 |
| The Sun | 8 Aug 2020 | ORGANS FOR SALE Kidney trafficker brags to The Sun about luring poor victims into selling organs to desperate Brits on Facebook for £85k | Negative | Negative | 1069 | 48 |
| Daily Star | 10 Aug 2020 | Formula One grid girl Khloe Atkinson dies after desperate kidney transplant plea | Positive | N/A | 348 | 16 |
| Daily Star | 12 Aug 2020 | Girl, 11, killed after being hit by car in horror crash as heartbroken family pay tribute | Positive | N/A | 348 | 16 |
| The Metro | 12 Aug 2020 | ‘Kind and caring’ girl, 11, dies two days after being knocked down in hit and run | Positive | N/A | 328 | 40 |
| The Sun | 12 Aug 2020 | HIT-&-RUN HORROR Girl, 11, killed in hit-and-run crash while crossing road with friend in Bury | Positive | N/A | 1069 | 48 |
| The Mirror | 22 Aug 2020 | Miracle transplant patient whose organs were ‘kept alive in a box’ set for dream wedding | Positive | N/A | 906 | 81 |
| The Mirror | 25 Aug 2020 | Brave Thalia, 5, dies waiting for heart transplant as heartbroken family pays tribute | Neutral | Neutral | 906 | 81 |
| Bishops Stortford Independent | 29 Aug 2020 | Bishop Stortford man, Stephen Cooper, the 100^th^ multi-organ patient at Addenbroke’s, salutes the surgeons and donor’s family who gave him a new life | Positive | Positive | 1 | 1 |
| ITV news | 1 Sept 2020 | The journey of organ donation: “Your son’s kidney saved my life” | Positive | Neutral | 924 | 120 |
| On the Wight | 4 Sept 2020 | Letter: Organ donation is now opt-out: let family know your wishes | Positive | Positive | 2 | 6 |
| Island Echo | 7 Sept 2020 | Local transplant recipient calls for families to talk about organ donation | Positive | Neutral | 9 | 4 |
| Northamptonshire Telegraph | 7 Sept 2020 | Kettering couple meet the boy who was given the gift of life by their daughter | Positive | Positive | 7 | 11 |
| Daily Express | 8 Sept 2020 | How to opt-out of being an organ donor | Positive | Neutral | 1351 | 50 |
| Daily Mail | 8 Sept 2020 | GP reveals how she offered to give her desperately ill baby son half of her own liver to save his life after struggle to find an organ donor – as she urges BAME community not to ‘opt out’ of register | Positive | Positive | 3256 | 117 |
| Northampton Chronicle and Echo | 8 Sept 2020 | Northampton’s oldest transplant patient urges public to sign organ donation register after surviving for 30 years with new heart | Positive | Positive | 8 | 11 |
| The Metro | 8 Sept 2020 | Family camps by bedside of girl, 1, in hospital waiting for a heart transplant | Positive | Neutral | 328 | 40 |
| Evening Standard | 9 Sept 2020 | Organ transplant waiting list jumps to five-year high due to pandemic, new NHS figures show | Positive | Positive | 282 | 64 |
| Asian Image | 10 Sept 2020 | New website aims to give Muslims the facts about organ donation | Positive | Positive | 2 | 7 |
| The Mirror | 11 Sept 2020 | Organ transplants among BAME patients at record highs after Mirror campaign | Positive | Positive | 906 | 81 |
| The Telegraph | 11 Sept 2020 | My scars aren’t ‘sexual’ – so why did Instagram remove my pictures? | Positive | N/A | 609 | 107 |
| The Times | 11 Sept 2020 | Minorities more likely to opt out of organ donation | Positive | Neutral | 357 | 117 |
| Yorkshire Post | 11 Sept 2020 | Organ Donation Week – meet the Yorkshire mum whose husband saved three lives | Positive | Neutral | 16 | 28 |
| The Metro | 13 Sept 2020 | Muslims encouraged to learn more about organ donation to help lack of donors for BAME people | Positive | Neutral | 328 | 40 |
| Plant based news | 14 Sept 2020 | Vegan TikTok star blasted for refusing to donate organs to meat eaters | Neutral | N/A | 5 | 95 |
| The Mirror | 27 Sept 2020 | Over half of pupils back organ donation after being inspired by Mirror campaign | Positive | Positive | 906 | 81 |
| Daily Star | 11 Oct 2020 | Fiancée of footballer, 25, who died in pub row gives birth to his baby daughter | Positive | N/A | 348 | 16 |
| Somerset Live | 16 Oct 2020 | 29 Somerset people on the organ transplant waiting list have died in the past five years | Positive | N/A | 16 | 11 |
| The Mirror | 18 Oct 2020 | Couple who lost baby son after he received a new heart in favour of organ donation | Positive | Positive | 906 | 81 |
| The Mirror | 21 Oct 2020 | Mum, 26, desperate for life-saving transplant to see her girl, 5, grow up | Positive | Positive | 906 | 81 |
| Worcester News | 3 Nov 2020 | Organ Donation Memorial gathering at Royal Worcestershire | Positive | N/A | 10 | 7 |
| Exmouth Journal | 7 Nov 2020 | Socially distanced photo opportunity as ‘opt in’ organ donation law comes into force | Positive | Positive | 1 | 3 |
| Daily Express | 9 Nov 2020 | New drive for donors as demand for organs reaches five year high | Positive | Positive | 1351 | 50 |
| Nursing Times | 9 Nov 2020 | ‘2020 is the year of talking about organ donation’ | Positive | Positive | 13 | 79 |
| BBC news | 20 Nov 2020 | Max and Keira’s law: mum’s ‘one comfort’ after son’s organ donation | Positive | Positive | 7781 | 152 |
| Birmingham Mail | 20 Nov 2020 | Nurse’s pride as son, 18, saves three lives after law change | Positive | Positive | 141 | 22 |
| The Mirror | 20 Nov 2020 | Heartbroken mum donates tragic son’s organs in historic first to save three lives | Positive | Positive | 906 | 81 |
| The Telegraph | 23 Nov 2020 | ‘My life was on old’: how patients awaiting transplants were hit by the closure of units | Positive | N/A | 609 | 107 |
| The Mirror | 30 Nov 2020 | Boy, 9, with three hearts says he can’t wait for Christmas after second transplant | Positive | Positive | 906 | 81 |
| Eastbourne Herald | 4 Dec 2020 | Organ donation gives people the gift of life and their freedom back | Positive | Positive | 5 | 9 |
| Yorkshire Evening Post | 4 Dec 2020 | Man born with one kidney is campaigning to keep organ donor register open during pandemic | Positive | Positive | 20 | 38 |
| Daily Echo | 10 Dec 2020 | Bournemouth family issues organ donation appeal to save son’s life | Positive | N/A | 31 | 6 |
| The Mirror | 10 Dec 2020 | “I almost died last Christmas – now I’ll celebrate this one twice with two of everything” | Positive | N/A | 906 | 81 |
| The Sun | 21 Dec 2020 | WORST NIGHTMARE Mum whose two children need life-saving kidney transplants is a match for both – but can only donate one | Positive | N/A | 1069 | 48 |
| The Telegraph | 24 Dec 2020 | Ten ways to give to charity without spending a penny | Positive | N/A | 609 | 107 |
| The Mirror | 25 Dec 2020 | Girl asks Santa for new heart for sister, 4, who has already lost half her foot | Positive | Positive | 906 | 81 |
| The Metro | 26 Dec 2020 | Dad who needs transplant to save his life urges black and Asian people to be organ donors | Positive | Neutral | 328 | 40 |
| The Mirror | 26 Dec 2020 | Woman whose life depended on double lung transplant and new heart gets Christmas miracle | Positive | Positive | 906 | 81 |
| The Metro | 31 Dec 2020 | Woman finally receives two new organs after first seven attempts went wrong at the last minute | Positive | Positive | 328 | 40 |
| Daily Mail | 2 Jan 2021 | How many like Natasha Tiwari have to die before Britain tackles its black and Asian organ donor crisis? Singer loses her fight for life five years after pleading for a new kidney | Positive | Positive | 3256 | 117 |
| BBC news | 7 Jan 2021 | Organ donor mum wishes she could help her children in need of kidneys | Positive | N/A | 7781 | 152 |
| The Telegraph | 13 Jan 2021 | Organs ready for donation discarded as transplant centres close due to pandemic | Positive | N/A | 609 | 107 |
| The Mirror | 18 Jan 2021 | Little Evie, 8, gets life-saving transplant after only match in the world drops out | Positive | Positive | 906 | 81 |
| The Mirror | 20 Jan 2021 | Schoolgirl saved by heart transplant gets another ‘perfect gift’ from donor’s parents | Positive | Positive | 906 | 81 |
| The Times | 23 Jan 2021 | Meet the people changing the world of organ transplants | Positive | Positive | 357 | 117 |
| Daily Mail | 26 Jan 2021 | Teenager who received a life-saving heart transplant reveals her donor’s grieving family have sent her a ring belonging to their 19-year-old daughter | Positive | N/A | 3256 | 117 |
| The Mirror | 30 Jan 2021 | My son was beaten to death at a bus stop – now I listen to his heart beat in a stranger | Positive | N/A | 906 | 81 |
| Daily Star | 7 Feb 2021 | Heartbreak as teenager, 19, dies in ‘freak accident’ after going to bed with a headache | Neutral | N/A | 348 | 16 |
| Daily Express | 10 Feb 2021 | Mother’s hope that daughter, 17, who donated organs, will inspire others | Positive | Positive | 1351 | 50 |
| Eastern Eye | 10 Feb 2021 | Donor family joins NHS campaign on organ donation | Positive | Positive | 3 | 45 |
| Telegraph and Argus | 10 Feb 2021 | The gift of life: Bradford backs campaign urging people to talk about organ donation | Positive | Positive | 40 | 9 |
| The Mirror | 10 Feb 2021 | Mum’s sudden decision weeks before her death saved four people’s lives | Positive | Positive | 906 | 81 |
| The Cumberland News | 11 Feb 2021 | NHS organ donor campaign is launched to get people talking | Positive | Neutral | 15 | 6 |
| Positive news | 12 Feb 2021 | Life-saving small talk: the hairdressers trained to chat about organ donation | Positive | Neutral | 2 | 102 |
| Cambridge Network | 15 Feb 2021 | Plea to leave no uncertainty on organ donation | Positive | Positive | 1 | 3 |
| The Metro | 18 Feb 2021 | Scientists regrow and repair liver from lab-grown cells in world first | Neutral | N/A | 328 | 40 |
| The Telegraph | 18 Feb 2021 | Lab-grown tissue repairs human organ for first time, Cambridge University scientists reveal | Neutral | N/A | 609 | 107 |
| BBC news | 21 Feb 2021 | Child heart transplants: record year for new-style operations | Positive | N/A | 7781 | 152 |
| Daily Mail | 21 Feb 2021 | ‘World first’ as NHS doctors transplant hearts in children using organs brought back to life by ground-breaking machine that replicates conditions inside human body | Positive | N/A | 3256 | 117 |
| Evening Standard | 21 Feb 2021 | Children given reanimated hearts by NHS in world first | Positive | N/A | 282 | 64 |
| iNews | 21 Feb 2021 | UK teens given reanimated hearts in world-first transplants | Positive | N/A | 119 | 57 |
| The Guardian | 21 Feb 2021 | UK doctors pioneer use of ‘heart in a box’ transplant technique in children | Positive | N/A | 3046 | 148 |
| The Metro | 21 Feb 2021 | Children receive hearts ‘brought back to life’ by NHS transplant machine | Positive | N/A | 328 | 40 |
| The Mirror | 21 Feb 2021 | World first as NHS saves six children by making ‘dead’ donor hearts beat again | Positive | N/A | 906 | 81 |
| The Telegraph | 21 Feb 2021 | Hearts brought back to life by ground-breaking machine given to children in world first | Positive | N/A | 609 | 107 |
| Evening Standard | 24 Feb 2021 | Woman dies after receiving ‘double lung transplant from donor with Covid-19’, report finds | Negative | N/A | 282 | 64 |
| The Mirror | 24 Feb 2021 | Max Johnson says “I did not think I would make it” in moving film about transplant | Positive | Positive | 906 | 81 |
| Shropshire Star | 26 Feb 2021 | Family whose son died in a car accident back organ donation campaign | Positive | N/A | 16 | 13 |
| The Mirror | 8 March 2021 | “I’d given up all hope and I knew I was going to die – then a phone call changed my life” | Positive | N/A | 906 | 81 |
| St Helen’s Star | 10 March 2021 | Violet-Grace posthumously recognised for organ donation which saved two lives | Positive | N/A | 5 | 7 |
| Daily Mail | 11 March 2021 | Snapchat partners with NHSBT to launch a body-tracking lens that lets you view your ORGANS through augmented reality | Positive | N/A | 3256 | 117 |
| Yorkshire Live | 11 March 2021 | Harrogate man ‘forever grateful’ to donor who saved his life | Neutral | N/A | 81 | 20 |
| Campaign | 12 March 2021 | NHS partners Snapchat to drive up organ donation awareness | Positive | N/A | 7 | 7 |
| Hampshire Chronicle | 12 March 2021 | Hampshire Hospitals backs organ donation campaign | Positive | Positive | 5 | 2 |
| Gazette and Herald | 13 March 2021 | Organ donation campaign launch | Neutral | N/A | 4 | 1 |
| Nursing Times | 15 March 2021 | Nurse recognised for work on organ donation law change during COVID-19 | Positive | Positive | 13 | 79 |
| Jewish Chronicle | 21 March 2021 | Jewish doctors launch survey to raise awareness of organ donation issues | Positive | Neutral | 5 | 1 |
| The Mirror | 30 March 2021 | Teen declared dead ‘miraculously’ starts blinking just before his organs were donated | Negative | N/A | 906 | 81 |
| The Mirror | 4 April 2021 | Transplant tots are hidden victims of pandemic as they desperately await life-saving ops | Positive | N/A | 906 | 81 |
| Daily Star | 6 April 2021 | Footballer Jordan Sinnott ‘saves seven lives’ with organ donations months after tragic death | Positive | N/A | 348 | 16 |
| The Metro | 11 April 2021 | My sister died in front of me but there was nothing I could do | Positive | N/A | 328 | 30 |
| The Mirror | 22 April 2021 | Loving lad hit by uninsured driver saves three lives after he’s robbed of his own | Positive | N/A | 906 | 81 |
| The Mirror | 9 May 2021 | “Mum’s dying gift means she lives on through my amazing new friends” | Positive | Neutral | 906 | 81 |
| The Sun | 13 May 2021 | ‘PERFECT SON’ Dad of Jordan Banks, 9, killed by lightning, says he had the ‘biggest heart’ and his donated organs helped save 3 kids | Positive | N/A | 1069 | 48 |
| Daily Express | 14 May 2021 | Boy killed by lightning saves three children by donating organs | Positive | N/A | 1351 | 50 |
| Daily Star | 14 May 2021 | Organs of boy killed in lightning strike have saved three other children’s lives | Positive | N/A | 348 | 16 |
| Evening Standard | 14 May 2021 | Jordan Banks: Boy who died when he was ‘struck by lightning’ to save three others after organ donation | Positive | N/A | 282 | 64 |
| The Metro | 14 May 2021 | Family of boy, 9, killed by lightning donate his organs to save three other kids | Positive | N/A | 328 | 40 |
| Sky news | 14 May 2021 | Jordan Banks: Boy who died after being ‘struck by lightning’ was organ donor and will save three lives, dad reveals | Positive | N/A | 538 | 413 |
| The Sun | 17 May 2021 | A TRAGIC nine-year-old boy died in hospital an hour after he was struck by lightning as he played football, an inquest heard today | Positive | N/A | 1069 | 48 |
| The Mirror | 19 May 2021 | Athlete saved by mystery heart donor after being on life support for nine days | Positive | Positive | 906 | 81 |
| BBC news | 20 May 2021 | 296 people donate organs in first year of new consent law | Positive | Positive | 7781 | 152 |
| The Metro | 20 May 2021 | Hospital carries out three organ transplants in just 17 hours | Positive | Positive | 328 | 40 |
| The Mirror | 20 May 2021 | Max and Keira’s 300 lifesavers who gave organs since donation law change one year ago | Positive | Positive | 906 | 81 |

| ***Full list of comments on media articles and tone*** | | | | | | | | | | |
| --- | --- | --- | --- | --- | --- | --- | --- | --- | --- | --- |
| **Publication** | **Date** | **Headline** | **Organ donation tone** | | | | **Law change tone** | | | |
|  |  |  | Article | Positive | Neutral | Negative | Article | Positive | Neutral | Negative |
| Daily Mail | 18 July 2019 | People dying fatter and older is ‘reducing the number of usable donated organs’ as NHS reveals one in SIX body parts now get rejected by doctors | Positive | 3 | 0 | 20 | Positive | 0 | 0 | 8 |
| Daily Mail | 20 Dec 2019 | Moving TV advert encourages families to talk about organ donation this Christmas before the law changes to make EVERY adult a donor next year | Positive | 14 | 1 | 12 | Positive | 11 | 1 | 23 |
| Daily Mail | 22 Dec 2019 | World’s first human HEAD transplant ‘could happen in next ten years’ due to advances in technology, says ex-NHS neurosurgeon and robotics expert | Negative | 4 | 0 | 11 | N/A | 0 | 0 | 0 |
| Daily Mail | 17 Feb 2020 | With a new opt-out donation law weeks away… DR MARTIN SCURR and DR MAX PEMBERTON question if the NHS should have the right to take our organs? | Positive | 20 | 0 | 13 | Neutral | 15 | 0 | 32 |
| Daily Mail | 31 March 2020 | Every organ donor is being tested for coronavirus as NHS rejects transplants from infected patients to minimise risk to recipients | Neutral | 3 | 0 | 1 | N/A | 0 | 0 | 2 |
| Daily Mail | 3 April 2020 | NHS bosses admit ALL organ transplants could be scrapped ‘within days’ over fears patients will catch coronavirus as outbreak overwhelms intensive care units | Positive | 7 | 0 | 3 | Neutral | 0 | 1 | 1 |
| Daily Mail | 13 April 2020 | Nursery worker, 21, who was waiting for life-saving liver operation dies after being taken off transplant list when she tested positive for coronavirus in hospital | Positive | 2 | 0 | 0 | N/A | 0 | 0 | 0 |
| The Metro | 20 May 2020 | From today every adult in England is automatically an organ donor | Positive | 19 | 4 | 8 | Positive | 18 | 1 | 24 |
| The Metro | 20 May 2020 | The new organ donation law will save lives, but it’s pointless if your family don’t know your wishes | Positive | 0 | 0 | 1 | Positive | 0 | 0 | 1 |
| The Times | 20 May 2020 | Every adult automatically an organ donor by law | Positive | 10 | 0 | 1 | Positive | 36 | 0 | 46 |
| Daily Mail | 21 May 2020 | I admit it, I feel queasy about the State having a claim on my organs… unless I choose to opt out, writes Melanie McDonagh | Positive | 26 | 0 | 11 | Negative | 15 | 0 | 46 |
| Daily Mail | 6 June 2020 | We’re all organ donors now – unless we opt out. Katie Hind’s moving story about her father’s incurable disease and how a new heart saved his life shows why the new law is so essential | Positive | 3 | 0 | 1 | Positive | 3 | 0 | 5 |
| On the Wight | 4 Sept 2020 | Letter: Organ donation is now opt-out: let family know your wishes | Positive | 6 | 0 | 4 | Positive | 3 | 0 | 6 |
| Daily Mail | 8 Sept 2020 | GP reveals how she offered to give her desperately ill baby son half of her own liver to save his life after struggle to find an organ donor – as she urges BAME community not to ‘opt out’ of register | Positive | 2 | 4 | 1 | Positive | 0 | 0 | 1 |
| The Times | 11 Sept 2020 | Minorities more likely to opt out of organ donation | Positive | 3 | 0 | 1 | Neutral | 1 | 0 | 2 |
| The Mirror | 27 Sept 2020 | Over half of pupils back organ donation after being inspired by Mirror campaign | Positive | 1 | 0 | 0 | Positive | 1 | 0 | 1 |
| The Mirror | 20 Nov 2020 | Heartbroken mum donates tragic son’s organs in historic first to save three lives | Positive | 6 | 0 | 0 | Positive | 1 | 0 | 1 |
| The Mirror | 18 Jan 2021 | Little Evie, 8, gets life-saving transplant after only match in the world drops out | Positive | 3 | 0 | 0 | Positive | 1 | 0 | 0 |
| The Times | 23 Jan 2021 | Meet the people changing the world of organ transplants | Positive | 12 | 0 | 0 | Positive | 2 | 0 | 0 |
| Daily Mail | 21 Feb 2021 | World first as NHS saves six children by making ‘dead’ donor hearts beat again | Positive | 43 | 1 | 20 | N/A | 0 | 0 | 8 |
| The Mirror | 20 May 2021 | Max and Keira’s 300 lifesavers who gave organs since donation law change one year ago | Positive | 2 | 0 | 2 | Positive | 2 | 0 | 2 |
