# Supplementary material for: Analysis of content and online public responses to media articles that raise awareness of the opt-out system of consent to organ donation in England
Source: Front Public Health. 2022 Dec 1;10:1067635. doi: 10.3389/fpubh.2022.1067635 (PMC9751921; doi:10.3389/fpubh.2022.1067635)
Supplement: Supplementary file 3 [file Table_3.DOCX]

- children
- desperate
- heart breaking
- tragedy
- rare condition
- miracle
- hope
- pride
- comfort
- memory
- hero
- culture
- taboo
- religion
- misinformation
- social media
- reciprocity
- trust
- racism
- autonomy
- individual rights
- control
- authoritarian
- dystopian
- undemocratic
- mandatory donation
- definition of death
- euthanasia
- organ transplant causing death
- selling organs
- organ farming
- harvesting
- transplants stopped
- fall in transplants
- Covid-19 pandemic
- role of healthcare workers
- lack of funding
- serious incidents
- breakthrough
- experimental
- animal organs
- unnatural
- science fiction
- Frankenstein
- playing god
- contamination
- Covid-19
- HIV
- drug users
- older people
- obesity
- fewer trauma incidents
