# Supplementary figures and images for: Analysis of content and online public responses to media articles that raise awareness of the opt-out system of consent to organ donation in England
Source: Front Public Health. 2022 Dec 1;10:1067635. doi: 10.3389/fpubh.2022.1067635 (PMC9751921; doi:10.3389/fpubh.2022.1067635)

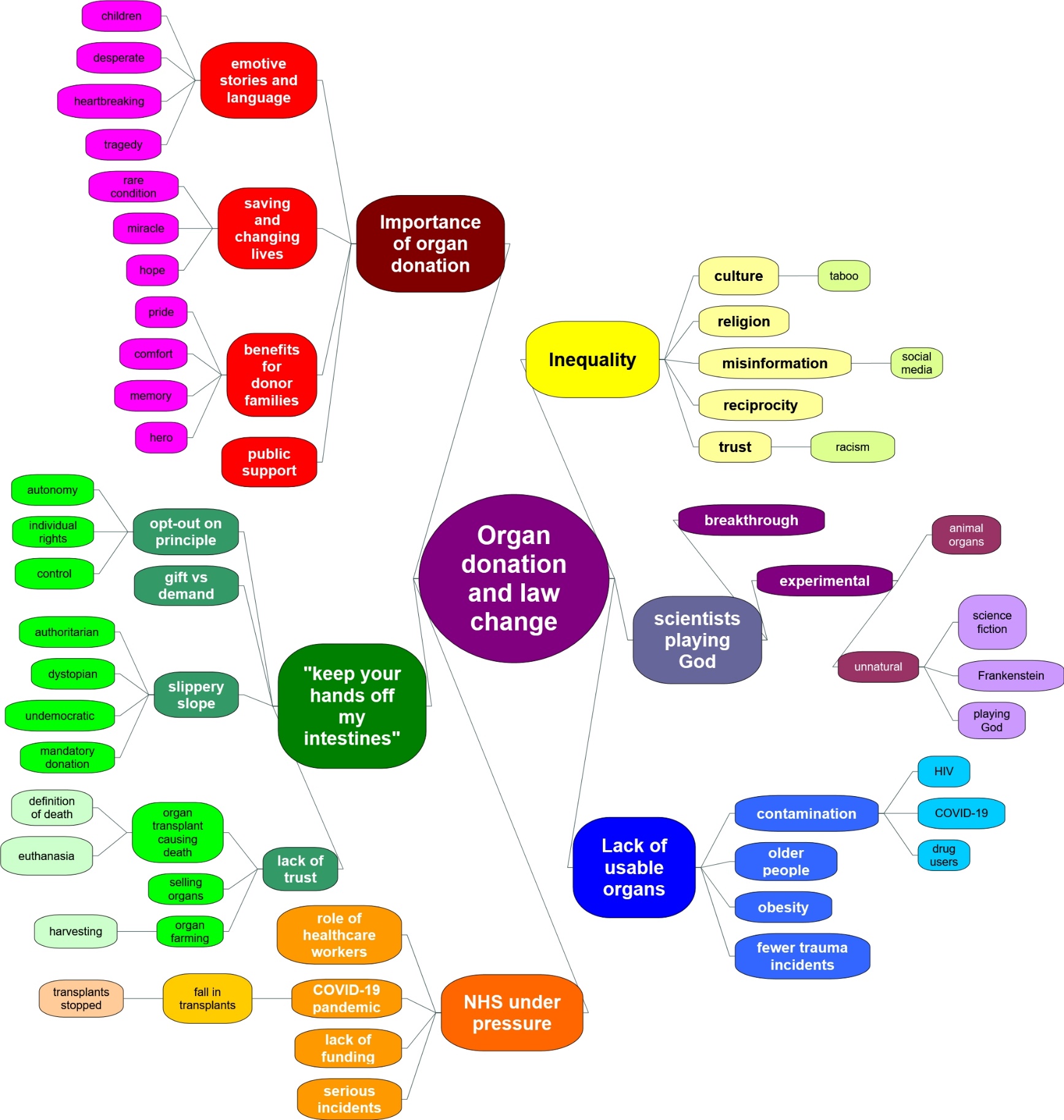

Supplement: Supplementary file 4 [file Table_4.DOCX]
